# The Role of Chronic Inflammation in Pediatric Cancer

**DOI:** 10.3390/cancers17010154

**Published:** 2025-01-06

**Authors:** Christine Mella, Panogiotis Tsarouhas, Maximillian Brockwell, Hope C. Ball

**Affiliations:** 1 Division of Hematology Oncology, Akron Children’s Hospital, One Perkins Square, Akron, OH 44308, USA; cmella@akronchildrens.org; 2 Department of Biology, The University of Akron, 302 Buchtel Common, Akron, OH 44325, USA; pan41@uakron.edu; 3 College of Medicine, Northeast Ohio Medical University, 4029 State Route 44, Rootstown, OH 44272, USA; mbrockwell@neomed.edu; 4 Rebecca D. Considine Research Institute, Akron Children’s Hospital, One Perkins Square, Akron, OH 44308, USA

**Keywords:** cancer, inflammation, epigenetics, therapeutics, immunology

## Abstract

Inflammation plays a crucial role in wound healing and the host immune response following pathogenic invasion. However, unresolved chronic inflammation can result in tissue fibrosis and genetic alterations that contribute to the pathogenesis of human diseases such as cancer. Recent scientific advancements exploring the underlying mechanisms of malignant cellular transformations and cancer progression have exposed significant disparities between pediatric and adult-onset cancers. For instance, pediatric cancers tend to have lower mutational burdens and arise in actively developing tissues, where cell-cycle dysregulation leads to gene, chromosomal, and fusion gene development not seen in adult-onset counterparts. As such, scientific findings in adult cancers cannot be directly applied to pediatric cancers, where unique mutations and inherent etiologies remain poorly understood. Here, we review the role of chronic inflammation in processes of genetic and chromosomal instability, the tumor microenvironment, and immune response that result in pediatric tumorigenesis transformation and explore current and developing therapeutic interventions to maintain and/or restore inflammatory homeostasis.

## Simple Summary

Chronic inflammation is associated with the onset and progression of many non-human diseases including type-2 diabetes, coronary artery disease, and cancer. The effects of chronic inflammation in adult cancers are widely studied. However, pediatric cancers demonstrate significant genetic/epigenetic disparities from their adult counterparts, requiring separate classifications. Because of this, findings from adult malignancies cannot be applied to pediatrics cancers. In this review, we examine how chronic inflammation contributes to the unique genetic and epigenetic changes, tumor microenvironment, and immune response that underly pediatric cancers. Finally, we highlight current and developing therapies aimed at restoring inflammatory balance during and after treatment.

## 1. Introduction

Inflammation is a central biological element of host defense and immunosurveillance [1,2]. Acute inflammation is beneficial to the local innate immune response following cell and tissue injury or pathogenic invasion. The inflammatory cascade is activated by tissue-resident macrophages and mast cells leading to localized vasodilation and the secretion of proinflammatory mediators. These mediators recruit neutrophils and monocytes that clear cellular debris and assess the need for a larger adaptive immune response [3,4,5]. Resolution of this localized pro-inflammatory state is a complex, step-wise, and tightly managed process. Activated immune cells either undergo apoptosis or transform to an inactive “sentinel” state, infiltrating neutrophils undergo efferocytosis, and there is an increase in secretion of pro-resolving and anti-inflammatory mediators [6,7,8,9]. Dysregulation in any or all of these resolving processes can result in a chronic state of inflammation that damages affected cells and tissues.

Chronic inflammation is associated with the pathophysiology of many non-communicable diseases, such as metabolic syndrome, type-2 diabetes mellitus, coronary artery disease, neurodegenerative diseases, and many types of cancer [10,11,12,13,14,15]. Extended exposure to pro-inflammatory mediators, such as chemokines, cytokines, and eicosanoids, can help promote the immune response to curb further tumor advance. However, chronic exposure also negatively impacts the healthy immune response and microenvironment through the disruption of cell-cycle checkpoints and interference with DNA repair promoting tumor growth and progression [16,17,18,19]. Chronic inflammation also contributes to pathological epigenetic changes, resulting in chromosomal abnormalities and aberrant histone modifications [16,17,18,19]. Together, these inflammation-associated biologic and epigenetic alterations have been shown to contribute to the onset and progression of disease.

Cancer is a multifaceted disease that is a major worldwide socioeconomic health problem and the second leading cause of death in the United States [20,21,22,23]. In adults, common risk factors include carcinogenic exposure, age, lifestyle, obesity, genetic predisposition, viral and/or bacterial infection, and chronic inflammation, a known comorbidity in colorectal, pancreatic, lung, lymphoma, breast, and cervical cancers [18,24,25,26,27,28,29]. Over time, repeated damage from these factors can result in genetic instability, epigenetic, and microenvironmental alterations that contribute to tumor cell carcinogenesis, proliferation, and metastasis [30,31,32].

Recently, scientific advancements have helped characterize the underlying mechanisms of many of these alterations and have exposed significant disparities between pediatric and adult-onset cancers [17,33,34]. In children and adolescents, cancer stands as the second leading cause of mortality [17,23,35,36]. Unlike their adult counterparts, pediatric cancers tend to harbor a lower overall mutational burden and arise in actively developing tissues targeting primarily undifferentiated stem or progenitor cell populations [37,38]. Mutations or dysregulations in the cell-cycle of these cell populations lead to the development of fusion genes (rarely detected in adult-onset cancers), epigenetic dysregulation, and pro-oncogenic changes to the tissue microenvironment [39,40,41]. The result of these genetic alterations is a sub-population of malignantly transformed cells with a stem-like phenotype that contribute to tumor growth, disease progression, and therapeutic resistance [42,43,44].

**Figure 1:**
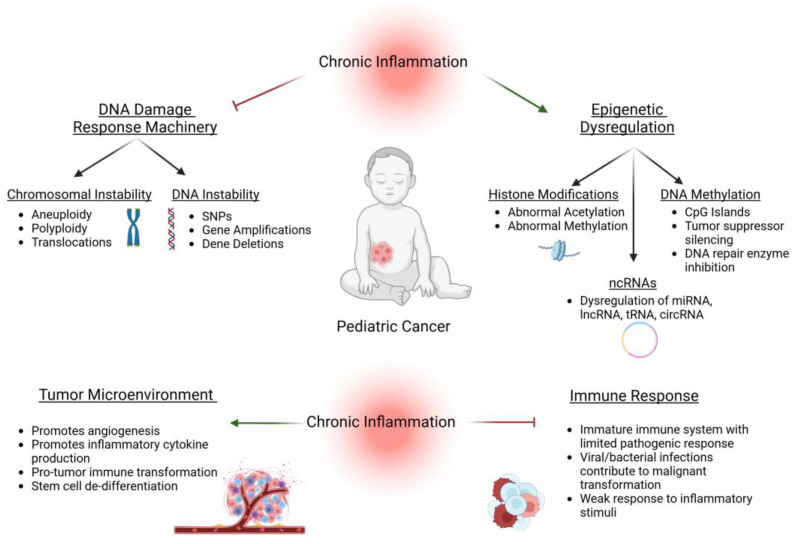
Chronic inflammation in pediatric cancer. Chronic inflammation plays a unique role in pediatric cancer, targeting differentiating cells and tissues. Alterations in the normal regulation of DNA repair, and abnormal epigenetic and chromosomal regulation alter the tumor microenvironment, promoting malignant transformation and tumor progression. The undeveloped and weakened pediatric immune system is limited in its ability to recognize and counter malignant cells contributing to cancer onset.

Many pediatric mutations are unique and their underlying etiology and molecular mechanisms remain poorly understood. Similarly, the role of chronic inflammation in pediatric tumorigenesis and therapeutic resistance has yet to be fully elucidated. This review will examine the role of inflammation in the processes of genetic and epigenetic instability, the tumor microenvironment, and immune response that result in pediatric malignant-transformation (Figure 1). Finally, we explore current and developing therapeutic interventions to maintain and restore inflammatory homeostasis during and after clinical treatment.

## 2. Materials and Methods

A PUBMED literature review was conducted to search for original papers on pediatric cancers and the role of chronic inflammation in cancer onset, progression, metastasis, drug resistance, and clinical outcomes. Key words included in the searches were “chronic inflammation and pediatric cancer”, “pediatric cancer and inflammation”, “chronic inflammation and genetic instability in pediatric cancer”, “genetic and epigenetic dysregulation in pediatric cancer”, “inflammation and epigenetic dysregulation in cancer”, “chronic inflammation and immune cells”, “chronic inflammation and the pediatric tumor microenvironment”, and “chronic inflammation and immune cells in pediatric cancer.” All figures were created with Biorender.com accessed on 4 December 2024.

## 3. Chronic Inflammation and Genetic Instability

**Figure 2:**
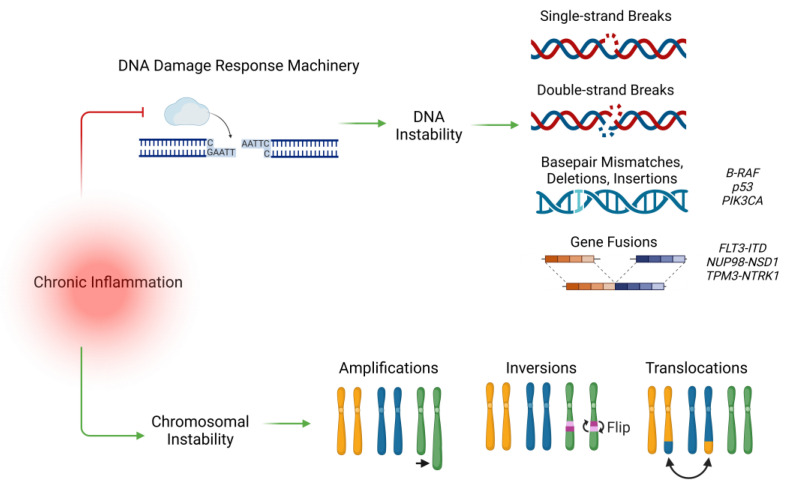
Chronic inflammation contributes to DNA and chromosomal instability. Chronic inflammation impairs the DNA damage response machinery resulting in the maintenance of oncogenic DNA breaks, splicing errors, and gene fusions. Exposure to chronic inflammation also contributes to the chromosomal abnormalities associated with pediatric cancer.

Genetic instability is a well-known hallmark of cancer [40,45]. Normally, instabilities are identified and managed by the DNA damage response machinery (DDRM), a network of cell-intrinsic and cell-extrinsic components that maintain genomic stability via efficient DNA repair, activation of cell-cycle checkpoints, or through induction of apoptosis and immune clearance of cells possessing unrepairable DNA damage [46,47,48]. In conditions of acute inflammation, these activities help to clear cells damaged by pathological infection and maintain cell and tissue homeostasis [48,49]. Chronic inflammation, however, inhibits the DNA response machinery, contributing to reduced onco-suppressive surveillance and increased risk of malignant abnormalities in both pediatric and adult cancers [50,51,52,53]. Inflammation-induced inhibition of the DNA damage response machinery results in transcriptional changes that negatively impact both chromosomal and genetic stability (Figure 2) [51,54,55].

Chromosomal instability, while rarer in pediatric cancers, is still an established contributing factor, particularly when defects alter the activity and/or function of oncogenes or tumor suppressors [56,57,58]. For instance, the gain or loss of a whole chromosome (aneuploidy) has been associated with the development of a variety of pediatric cancers including astrocytoma, B-cell acute lymphoblastic leukemia (ALL), optic tumors, neuroblastoma (NB), rhabdomyosarcoma, and osteosarcoma (OS) [59,60,61,62]. Similarly, the gain of whole chromosome sets (polyploidy) has been identified in pediatric oligodendroglioma, rhabdomyosarcoma, ALL, Ewing sarcoma, and testicular germ cell tumors [63,64,65,66]. Changes in chromosome structure are also prevalent in pediatric cancers leading to abnormal fusion, amplification, or chromosomal translocation [32,67]. Indeed, structural changes have been identified and linked to predicted clinical outcomes in astrocytoma, ALL, renal cell carcinoma, and several pediatric sarcomas such as Ewing sarcoma, OS, rhabdomyosarcoma, and synovial sarcoma [67,68,69,70,71]. Finally, inactivation or deletion of chromatin remodeling genes have also been shown to contribute to chromosomal instability. For instance, inactivating mutations in the SWI/SNF-related matrix-associated actin-dependent regulator of chromatin subfamily b member 1 (SMARCB1) have been identified in pediatric malignancies such as medulloblastoma (MB), atypical teratoid rhabdoid tumors (ATRT), and ovarian carcinomas, which impair cell differentiation and maintains the cancer stem cell-like phenotype [72,73,74,75].

The loss of normal DNA repair mechanisms following exposure to chronic inflammation also negatively impacts DNA stability and permits abnormal promoter defects, gene duplication, gene fusion, single nucleotide variants (SNVs), and gene copy number changes [48,76,77,78,79,80]. These germline variants, found in around 10% of pediatric cancer patients, can either be inherited or occur de novo [81,82]. Increases in genetic testing have identified a number of point mutations such as B-RAF (*BRAF^V600E^*), tumor protein P53 (*p53*), phosphatidylinositol-4,5-bisphosphate 3-kinase, catalytic subunit alpha (*PIK3CA*), Kirsten rat sarcoma virus (*KRAS*)*,* and catenin beta 1 (*CTNNB1*) [83,84,85,86,87,88,89,90,91]. Gene fusions have also been identified in pediatric cancers. Some of these, such as *FLT3–ITD*, *NUP98–NSD1,* and *TPM3–NTRK1* fusions in hematologic neoplasms, the *EWS–FLI1* fusion in Ewing sarcoma, and *PAX–FOXO1*, associated with rhabdomyosarcoma, have been clinically linked and functionally examined, but many still remain identified but unexplored [92,93,94,95,96,97,98]. Gene amplifications and deletions such as MYCN proto-Oncogene (*MYCN*), cyclin-dependent kinase inhibitor 2A (*CDKN2A*), mitogen-activated protein kinase (*MAPK*), epidermal growth factor receptor (*EGFR*), fibroblast growth factor receptor (*FGFR*), wingless (*WNT*), and sonic hedgehog (*SHH*) have been identified and are associated with risk stratification and prediction of clinical outcomes [99,100,101,102,103]. Finally, cancer cells themselves are known to directly contribute to the proinflammatory milieu and further DNA damage. Due to an elevated cellular metabolism and mitochondrial dysregulation, cancer cells generate free radicals such as reactive oxygen species (ROS) and reactive nitrogen species (RNS). Neoplastic cells also secrete proinflammatory cytokines, such as nuclear factor kappa-light-chain-enhancer of activated B (NF-κB), transforming growth factor beta 1 (TGFβ-1), and tumor necrosis factor alpha (TNF-α) [104,105,106,107,108,109]. Together, these compounds damage both DNA and RNA, contribute to lipid peroxidation, and activate signaling pathways governing angiogenesis, invasion, and metastasis [110,111,112,113].

## 4. Chronic Inflammation and Epigenetic Dysregulation:

**Figure 3:**
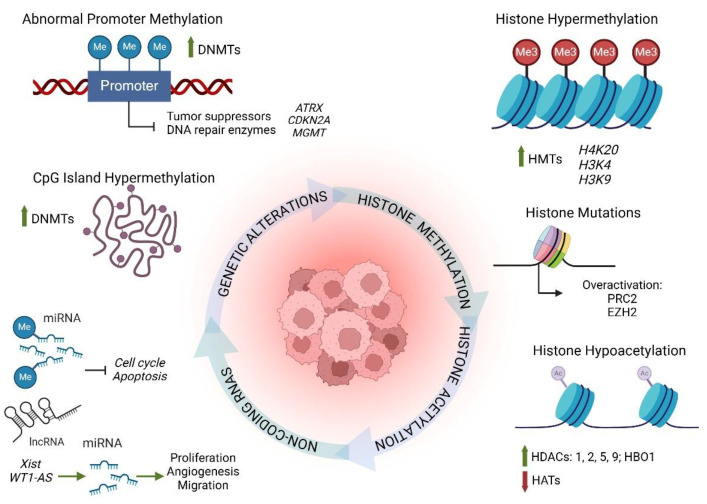
Chronic inflammation contributes to oncogenic genetic and epigenetic alterations. Exposure to chronic inflammation results in abnormal DNA and promoter methylation patterns that promote tumor growth and progression. Abnormal histone methylation and acetylation in malignant cells contribute to altered gene expression patterns affecting prognosis and drug response. Non-coding RNA alterations inhibit normal cell-cycle regulation and promote cancer cell migration.

Chronic inflammation plays a role in epigenetic dysregulation, which contributes to the pathogenesis of numerous diseases including osteoarthritis, pulmonary disease, metabolic diseases, and cancer [114,115,116,117]. In pediatric cancers, studies have not only identified many epigenetic modifications but have also demonstrated strong correlations between these modifications and drug response, progression, and overall predicted outcome [118,119,120,121]. These changes may be of either mitotic or meiotic in origin and alter epigenetic regulation of DNA access, histone access, or the activity of non-coding RNAs (Figure 3). These changes alter the degree and/or timing of pathways associated with differentiation, growth and specialization [122,123,124]. Of particular interest in developmentally based pediatric cancers, oncogenic epigenetic changes are known to affect genes, such as SRY (sex-determining region Y)-box 2 (*SOX2*), CD44 molecule (*CD44*), and prominin-1 (*CD133*), contributing to enhanced capacity for self-renewal and stem-cell-like phenotypes [40,125,126,127].

Chronic inflammation is known to contribute to changes in DNA methylation patterns in cancer, multiple sclerosis, and autoimmune diseases [128,129,130,131]. For example, adult dialysis patients demonstrate elevated inflammation markers and significantly higher global DNA methylation patterns compared to age-matched healthy controls [132]. Epigenetic profiling in the pediatric population has also uncovered elevated DNA methylation patterns associated with the development and progression of atopic asthma, Crohn’s disease, and ulcerative colitis [133,134,135,136]. The process of DNA methylation is a tightly regulated process in which enzymes known as DNA methyltransferases (DNMTs) add methyl groups to specific DNA locations [137]. DNA methylation alters the three-dimensional confirmation and modifies DNA access and the binding potential of various transcription factors and associated proteins [138].

DNA methylation is particularly important during development, where it assists in the tight regulation of gene expression, cell and tissue differentiation, and genomic stability [139]. One common dysregulation associated with pediatric cancers involves abnormal methylation of CpG islands. CpG islands are short regions of DNA, approximately 500–1000 base pairs in length, with an elevated percentage of cytosine–guanine dinucleotides [140,141]. CpG islands are common at or near promoter regions and under normal conditions are not usually methylated [50,142]. In pediatric cancer, however, genetic studies have uncovered abnormal CpG island methylation in patients with ALL, OS, gastric carcinoma, and brain tumors [51,143,144,145]. Abnormal methylation of CpG islands results in transcriptional silencing of key tumor suppressor gene promoters (such as alpha-thalassemia mental retardation X-linked (ATRX) and cyclin-dependent kinase inhibitor 2A (CDKN2A)) in addition to DNA repair enzymes (such as O6-methylguanine methyltransferase (MGMT)), resulting in abnormalities in the cell cycle, DNA repair, and apoptotic pathways [51,146,147]. Methylation status has also been linked to prognosis, drug resistance, and metastasis [148,149,150,151,152]. As such, therapeutic treatments targeting DMNTs are being used to reactivate these pathways and decrease overexpression of oncogenic targets in cancer cells [153,154,155].

Along with DNA methylation, transcriptional activity and timing are regulated via histone function. Histones are proteins enriched in the amino acids arginine and lysine, which organize DNA into nucleosomes to regulate gene function and protect against DNA damage [156,157]. There are five main core histone proteins (H1/H5, H2A, H2B, H3, and H4) responsible for establishing the tightly compacted nucleosome structure [158,159]. Post-translational modifications, such as methylation, acetylation, ubiquitination, and phosphorylation, further contribute to transcriptional control by inducing conformational change [160,161]. In cancer, epigenetic regulation of histone post-translational modifications is dysregulated, disrupting normal cell proliferation and differentiation [159,162,163]. Of the post-translational histone modifications associated with pediatric cancer, abnormalities in histone methylation and acetylation are the most widely studied.

Proper activity of histone-methylating enzymes is necessary for cell differentiation and tissue development. Histone methyltransferases (HMTs) are responsible for the addition of methyl groups, which can later be removed via the activity of histone demethylases (HDMTs) [112,164]. The effects of histone methylation are site-specific and relative to the degree of methylation, i.e., the addition of single or multiple methyl groups [161,165]. The degree of histone methylation can also be modified by inducible Jumonji enzymes. Jumonji domain-containing protein-3 (Jmjd3, also known as KDM6B) plays a critical role in epigenetic regulation of stem cell differentiation and cellular reprograming [166,167,168]. Exposure to bacterial proteins and inflammatory cytokines results in increased Jmjd3 expression, demonstrating a link between the inflammation and epigenetic alteration [169,170]. In the pathogenesis of pediatric cancers, multiple disruptions in histone methylation have been identified. For instance, a hypermethylation of histone 4 lysine 20 (H4K20) compared to levels detected in healthy tissue has been identified in some pediatric astrocytomas, ependymomas, and diffuse pontine gliomas [171,172,173,174]. H4K20 is of particular importance since it is known to regulate genes maintaining telomere length and governing epithelial–mesenchymal cell differentiation [175,176]. Furthermore, hypermethylation of H4K20 contributes to gene silencing in pediatric leukemia and NB that inhibit the DNA repair machinery and enhance cancer cell invasion into surrounding tissues [177,178,179]. Trimethylation of histone 3 lysine 4 (H3K4me3) has been identified in ependymomas and MB and been shown to correlate with both tumor grade and increased chemotherapeutic resistance [180,181,182]. Missense mutations in glycine 34 of histone 3 (H3G34) have been shown to directly contribute to the genetic instability and oncogenic expression profiles of pediatric gliomas, sarcomas, and bone cancers [181,183,184,185,186]. These mutations are frequently observed in pediatric cancers and function to impede SETD2 trimethylation of H3K36 to promote increased activity of polycomb group protein 2 (PRC2), which silences key cell-cycle regulators, impairs normal DNA damage responses, and increases proinflammatory cytokine production [187,188,189]. Increased methylation of histone 3 lysine 9 (H3K9) is associated with increased chromosomal instability, higher astrocytoma tumor grade and poor prognosis [174,190]. Mutations also occur in histone methyltransferases. One of the best studied examples is an overexpressing mutation in histone methyltransferase enhancer of zeste hologog 2 (EZH2), which has been identified in MB, gliomas, soft tissue carcinomas, sarcomas, rhabdoid tumors, and both B-cell and T-cell lymphoproliferative disorders [191,192,193,194]. Overexpression of EZH2 promotes overexpression of H3K27-specific demethylases, resulting in altered chromatin structure and reduced DNA damage response, and confers radiation resistance [40,195].

Histone acetylation, the addition of an acetyl group, also causes confirmation changes that can alter gene access and transcription. The addition of an acetyl group, catalyzed by histone acetyltransferases (HATs), loosen DNA–histone binding to permit gene access for transcription [196]. This process, like histone methylation, is tightly regulated and reversible. Acetyl groups can be removed from histones (or histone side chains) to inhibit transcription by histone deacetylases (HDACs), which are grouped into four classes based on structure, function, and location [197,198,199]. Abnormal histone acetylation or HAT/HDAC interactions with histone-associated co-factors are known to play a role in pediatric cancer and have been correlated with cancer onset, therapeutic response, and overall prognosis [124,200]. The elevated activity of HATs not only alters histone modification but is known to contribute to chronic inflammation via increased expression of pro-inflammatory cytokines such as interleukins 2, 8, and 12 (IL-2, IL-8, IL-12). NF-κB signaling, significantly upregulated in many pediatric cancers, also plays a role in the maintenance of abnormal histone acetylation, particularly histone 3, where acetylation promotes inflammatory chemokine and cytokine production [169,201,202,203,204]. Changes in the expression and activity of HATs and HDACs have been described in solid tumors, B-cell progenitor acute lymphoblastic leukemia, ALL, acute myeloid leukemia (AML), and OS [205,206,207,208]. An increase in histone 3 lysine 16 (H3K16) acetylation has been associated with diffuse intrinsic pontine gliomas, and high expression profiles of HDAC5 and HDAC9 have been associated with MB subgroups that typically present with high risk and poor overall survival [124,171,209]. Similarly, high expression profiles of HDAC1 and HDAC2 have been identified in cancer-associated fibroblasts of pediatric hepatoblastoma and hepatocellular carcinoma patients, which contributes to lung metastases and promotes elevated expression of p21, a known inhibitor of cell-cycle regulation that affects proliferation [210,211]. Cancer-associated gene mutations and amplifications also play a role in epigenetic dysregulation of histone acetylation. In childhood midline tract carcinomas, gene fusions in NUT midline carcinoma family member 1 (*NUTM1*) affect the activity and function of bromodomain-containing 4 (*BRD4)*, a chromatin scaffold protein with intrinsic histone acetyltransferase activity [212,213]. Overexpression of histone lysine acetyltransferase HBO1 (KAT7), which acetylates histones 3 and 4, is associated with OS cancer proliferation and enhanced cell migration [207,214]. In MB, amplifications of *MYCN* and MYCL proto-oncogene (*MYCL1*) have been identified and correlated with cancer aggressiveness and increased risk of metastasis [215,216]. This amplification can also dysregulate epigenetic regulation of histone acetylation via the recruitment of HATs, resulting in enhanced metabolism, proliferation, and viability of cancer cells [217,218]. CREB-binding protein, CREBBP, is a protein with intrinsic acetylase function known to add acetyl groups to not only histone residues but also various transcription factors [219]. In pediatric hematological malignancies, CREBBP is overexpressed compared to healthy controls and this overexpression is typically correlated with a higher risk stratification and poorer prognostic outcome [208,220]. Furthermore, studies have demonstrated that mutations in HAT expression and activity are associated with an increased chemotherapeutic resistance in both B-cell progenitor- and acute lymphoblastic-leukemias [205,208]. Finally, dysregulation of histone acetylation has been shown to directly influence the pro-inflammatory cancer microenvironment through hyperacetylation of pro-inflammatory transcription factors such as signal transducer and activator of transcription 1 and 3 (STAT1/3) and NF-κB [221,222].

Non-coding RNAs are another well-established means of epigenetic regulation in cancer. Regulatory function typically occurs via one of two methods: complementary base pairing to 3′ untranslated regions to inhibit translation or targeting mRNAs for degradation by direct complementary binding [223,224]. Non-coding RNAs are also a well-established player in the regulation of chronic inflammation, where they are known to regulate immune cell differentiation and development, the expression of pro- and anti-inflammatory cytokines, and the resolution of the acute inflammatory response [123,225,226]. Additionally, non-coding RNAs are capable of directly impacting the epigenetic landscape by targeting the activity of other epigenetic regulators, such as DNA and histone methyltransferases and deacetylases [227,228,229]. Non-coding RNAs comprise several classes based on size, function, and structure [123,225]. These include microRNAs (miRNAs), transfer RNAs (tRNAs), long non-coding RNAs (lncRNAs), and circular RNAs (circRNAs), among others. Studies of these classes have identified dysregulations associated with cancer and other human pathologies [230,231]. In developmental diseases such as pediatric cancer, lncRNAs and miRNAs play a large role in cell differentiation and tissue development [232,233]. For instance, lncRNA XIST, an X-chromosome inactivator that interacts with polycomb repressive complexes (PRC1/2) mediates the ubiquitination and methylation of histones to modulate gene expression during female development [234,235,236]. XIST is abnormally upregulated in pediatric brain cancers like gliomas, pituitary endocrine tumors, and neuroblastoma, where it interacts with various miRNAs to increase cell proliferation, migration, and angiogenesis, and inhibit apoptosis [237,238,239]. Similarly, abnormal miRNA interactions with lncRNA WT1-AS, a non-coding RNA that modulates developmental nephrogenesis and EZH2 suppression, have been shown to play a role in the pathogenesis and increased malignancy of Wilms’ tumors [123,240]. Other lncRNAs have also been found to be abnormally expressed and play an oncogenic role in cell proliferation, migration, progression, and drug resistance in lymphoblastic leukemia, OS, retinoblastoma, NB, and MB [241,242,243,244]. In Hodgkin’s lymphoma, a pediatric cancer with a well-studied pro-inflammatory milieu, recent studies have identified numerous dysregulated miRNAs [245,246,247]. Methylation studies have also uncovered abnormal methylation patterns that disrupt normal miRNA function in pediatric cancers. In NB and MB, activating methylation of miRNAs disrupts cell-cycle progression and apoptosis and enhances MYCN proto-oncogene function and increases cancer cell migration [248,249,250,251,252,253]. The function of tumor suppressor miRNAs is inhibited through enhanced methyltransferase activity in Hodgkin lymphoma, ALL, AML, and hepatocellular carcinoma [254,255,256]. Recent studies have also implicated miRNAs in the acquisition of chemotherapy resistance in OS, primary malignant brain tumors, and leukemias [257,258,259].

## 5. Chronic Inflammation and the Tumor Microenvironment

The tumor microenvironment (TME) of adult and pediatric cancers is complex, heterogenous, and dynamic. The TME includes a wide array of cell types including malignantly transformed cancer or cancer stem cells, non-cancerous stromal cell populations (such as endothelial cells, fibroblasts, pericytes, neuronal cells, and adipocytes), various types of infiltrating immune cells (such as T-cells, B-cells, macrophages, and dendritic cells), components of underlying and supportive extracellular matrix and vascular system, and various signaling molecules responsible for tissue homeostasis and inter-/intra-cellular communication [260,261].

Comprehensive molecular analyses of adult cancers have greatly improved our knowledge regarding the adult TME, particularly the various types of infiltrating immune cells and their biological roles in adult malignancies [262,263,264]. However, these findings cannot be applied directly to pediatric tumors due to numerous disparities in affected cell types, underlying genetic/epigenetic abnormalities, and developmental status between adult and childhood cancers [34,265,266]. As such, further research is needed to better elucidate the complex web of chemical and cellular signaling that drives pediatric cancer onset, progression, and therapeutic resistance.

Further complicating the direct use of adult studies in pediatric cases, there are developmental differences in pediatric immune systems, critical in the early detection and removal of malignant cells [267]. The adult immune system consists of B- and T-cells with extensive memories for antigen recognition following infection or pathogenic invasion [268,269,270,271]. Over time, fetal-derived immune cells and repeated exposure to pathogens, allergens, and vaccines builds a complex and varied immune system capable of responding to a wide variety of threats, including early detection of malignant cell types [272,273,274]. This is of importance since microorganisms are known to cause a significant number of adult cancers [275,276,277]. In childhood, however, this immune barrier has yet to be established and the immune system is comparatively weak. In newborns, macrophage and monocyte populations are immature and neutrophil responses to pathogens is weak [278,279,280,281]. This leaves newborns and infants more susceptible to bacterial and viral infections and reduces tissue repair mechanisms [282,283]. The weakened immune response to inflammatory stimuli also contributes to the pathogenesis of several childhood cancers. For instance, the Epstein–Barr herpesvirus (EB) has been linked to Burkitt lymphoma, Hodgkins’ disease, and cases of nasopharyngeal carcinoma [284,285,286]. This is thought to occur through the onset of genomic instability brought about by B-lymphocyte interactions with EB structural proteins [287,288]. Viral infection with human neurotrophic polyomavirus (JCV) has also been linked to the development of CNS tumors. Here, JCV T-antigens inhibit critical cell-cycle regulators such as p53 and retinoblastoma protein (pRb) contributing to CNS tumor onset and progression in humans and animal models [276,289,290,291].

**Figure 4:**
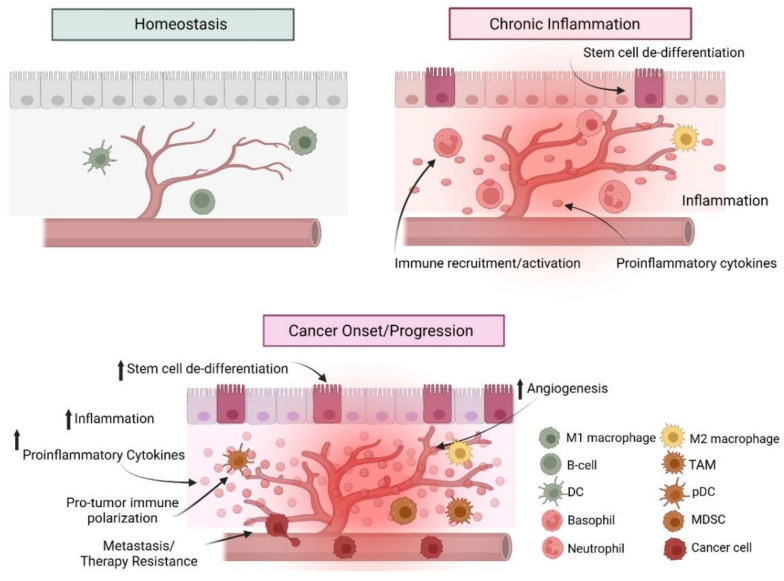
Chronic inflammation alters the tissue microenvironment and immune recruitment to promote tumor onset, progression, and metastasis. Chronic inflammation promotes de-differentiation and/or malignant transformation of cells within the microenvironment, increased angiogenesis, and proinflammatory cytokine release. Conditions within the tumor microenvironment (TME) promote metastasis and inhibit immune cell recognition and targeting of malignantly transformed cancer cells. Arrows indicate an increase.

Inflammation plays a pivotal role in immune cell recruitment to both healthy tissues and the TME. Following the onset of acute inflammation due to infection or tissue damage, various cytokines and acute proteins activate the innate immune response through the recruitment of neutrophils and macrophages [292,293]. If the infection or injury persists, antigen-presenting cells activate and recruit T-cells as part of the adaptive immune response [294,295]. Once the injury or infection is resolved, acute inflammation subsides. Chronic inflammation, however, is a prolonged response resulting in a damaging pro-inflammatory microenvironment and abnormal levels of immune cell infiltration. In the TME, cancer or cancer stem cells secrete pro-inflammatory agents, such as cytokines, chemokines, and prostaglandins, to maintain inflammation, promote angiogenesis and fibrosis, and foster an immunosuppression via deregulated immune cell function (Figure 4) [296,297,298,299]. Recently, studies have examined differences in the pediatric TME towards the goal of immunotherapy development. These studies have revealed pediatric cancer TMEs demonstrate distinct immuno-profiles that vary between tumor type, subtype, and stage, and correlate with identified underlying genetic and epigenetic abnormalities [300,301,302,303]. Specifically, many of these studies have examined alterations in infiltrating myeloid precursors, macrophages, and dendritic cells of the pediatric TME in relation to their biological function and clinical outcomes.

Myeloid precursors play a vital role in host defense against pathogens and in tissue repair following injury. These immature cells are capable of differentiating into a variety of cell types including macrophages, dendritic cells (DCs), and granulocytes [304,305]. However, in the TME upregulation of pro-inflammatory cytokines, such as IL-1β and IL-6, and prostaglandin E2 (PGE2) disrupts normal myeloid cell differentiation that results in an accumulation of immunosuppressive myeloid-derived suppressor cells (MDSCs) [304,306]. TME MDSCs promote angiogenesis via secretion of matrix metalloproteinase 9 (MMP9), maintain the inflammatory microenvironment through the generation of ROS and RNS, and inhibit T-cell function via secretion of arginase 1 (ARG1) [307,308,309]. Clinically, elevated MDSC levels are associated with pediatric gliomas, NBs, sarcomas, and leukemias where levels correlate with cancer stage, metastatic capacity, and overall survival [307,310,311,312].

Macrophages are an abundant heterogenous population of white blood cells that play crucial roles in development, host immune surveillance, intracellular communication, and the inflammatory response [313,314]. Macrophage origins vary depending on age and tissue of origin, deriving from hematopoietic stem cells (HSCs), monocytes, or tissue-resident macrophages of the fetal liver [315]. Macrophages are first recruited via secretion of macrophage colony-stimulating factor (M-CSF) and recruitment is enhanced through secretion of vascular endothelial growth factor (VEGF), TNF-α, and monocyte chemoattractant protein 1 (MCP-1; also known as CCL2 (C-C motif chemokine ligand 2) signaling [316,317]. Following recruitment, these cells are polarized to either an M1 or M2 state based on the regulatory molecules found in the tissue microenvironment. M1 macrophages are polarized by environmental cues such as interferon-gamma (IFN-γ) secreted by CD4^+^ T-helper or natural killer cells or TNF-α from macrophages and antigen-presenting dendritic cells [318,319]. M1-polarized macrophages secrete cytokines such as TNF-α and IL1-β and are known to upregulate major histocompatibility complex call 2 (MHC-II) to initiate CD4+ T-cell activation and coordinate immune cell activity [320,321]. Alternately, macrophages can also be polarized to the anti-inflammatory M2 phenotype. In the TME, M2 macrophages serve an immunosuppressive function via secretion of immune inhibitory cytokines such as TGF-β and interleukin-10 (IL-10) [305]. Also, M2 macrophages promote tumor growth and progression through secretion of angiogenic VEGF and growth factors such as platelet-derived growth factor (PDGF) and basic fibroblast growth factor (bFGF)) [322,323]. M2 macrophages have also been shown to inhibit cytotoxic CD8+ T-cell identification and clearance of tumor cells in adult cancer through surface expression of programmed cell death ligand 1 (PD-L1) [324,325]. Interestingly, in pediatric cancers, M2 expression of PD-L1 has been shown to be extremely low to non-existent, which may explain why current PD-1/PD-L1 immunotherapies demonstrate mixed results [312,326,327,328]. In pediatric cancers, the polarization state of infiltrating macrophages in the TME have been linked to prognosis, invasiveness, and risk of metastasis. For instance, a TME enriched with a higher proportion of M2 immunosuppressive macrophages has been shown to correlate with more aggressive and metastatic OS tumors and a poorer prognosis [329,330,331]. Conversely, TMEs with a higher percentage of M1 macrophages predict better therapeutic responses and overall survival in MB, NB, and pediatric gliomas [260,323,332].

Dendritic cells (DCs) are immune cells with a critical role in communication between the innate and adaptive immune systems [333,334]. Following injury or pathogen recognition, mature DCs are responsible for antigen presentation and subsequent activation of naïve T-cells to support anti-tumor T-cell maturation and function [305,335]. DCs are separated into two categories depending on location, expression profile, and phenotype: classic DCs (cDCs) and plasmacytoid DCs (pDCs) [336,337]. While the exact function of each type of DC is still being determined, studies have indicated that cDCs prime CD8+ T-cells for anti-tumor recognition while pDCs produce high levels of interferon gamma (INF-γ), which promotes an immunosuppressive TME [338,339,340]. Recent studies have examined DC prevalence within the TME of adult and pediatric malignancies and shown that DC infiltration is significantly lower in pediatric tumors [341]. Furthermore, studies have shown that DC maturation is inhibited in the TME of pediatric Ewings, ATRTs, gliomas, and hematologic malignancies to favor tumor growth and progression [342,343,344,345]. Given these findings and the crucial role of DCs in tumor recognition and anti-tumor host response, cancer vaccine and immunotherapy developments are underway utilizing DC as a potential vehicle [346,347].

The TME can also be altered during necrosis, unregulated cell death. Cells undergoing necrosis swell and release cellular contents into the surrounds tissue. Within a tumor, the core is typically populated with cells that have undergone necrosis and the presence of these areas are often associated with metastatic capacity and poor prognosis [348,349]. Necrotic cells release a potent mixture of damage-associated molecular patterns (DAMPs) that contribute to the chronic inflammatory milieu and promote tumor growth [350,351]. Furthermore, necrotic cells release high amounts of potassium during cell rupture, which inhibits the function of activated T-cells [352,353]. Also, necrosis can form as a result of chemotherapy in a variety of cancer types such as OS, NB, and ALL [354,355,356].

## 6. Conclusions

Pediatric cancer, while relatively rare, remains a serious public health concern. The most common childhood cancers between birth and early teens remain leukemia, central nervous system (CNS) cancers, and lymphoma [23,123]. The past few decades have seen a decline in overall mortality rates due to breakthroughs in diagnostic imaging and clinical treatments, improvements in genetic testing rates, and novel immunotherapies [76,77,357,358]. However, for pediatric bone and CNS cancers, incidence and overall survival has not changed significantly due to factors such as tumor accessibility, stage at diagnosis, underlying genetic alterations, and a unique stem-cell-like population of transformed cells that possess the means to modulate the TME to avoid immune detection [23,50]. Cancer-cell-mediated alterations to the TME often involve hijacking the host inflammatory response to promote tumor growth and progression.

The pathological switch from an acute inflammatory response to one of chronic inflammation contributes to the pathophysiology of numerous adult and pediatric diseases. The chronic, pro-inflammatory milieu of pediatric cancers promotes tumor growth and progression via secretion of angiogenic chemokines, growth factors, and suppression of the immune response [7,359]. Several key pro-inflammatory cytokines play roles in the pathogenesis of both adult and pediatric cancers. For instance, in adults, TNF-α, produced by immune cells such as lymphoid and macrophage populations, can inhibit T-cell response and activation of cancer cytotoxic T-call activation through interactions with its receptor TNFRSF1B (TNFR2) [360,361]. In pediatric cancer, TNF-α has been shown to promote de-differentiation of OS [362], Furthermore, the immature state of pediatric T-cells has been shown to contribute to reduced expression of CD40, a tumor necrosis factor receptor family member [363]. Known to promote M2 to M1 polarization in monocytes and macrophage populations, reduced CD40 expression in pediatric immune cells results in reduced anti-tumor response [364]. Interleukin 1 (IL-1) is another pro-inflammatory cytokine with known connections to the tumor microenvironment. IL-1 can activate NF-κB and MAP kinase pathways via the IL-1R1 receptor [365]. Expressed by cells of the innate and adaptive immune systems, the expression of this receptor in immune cells has been linked to cancer stages and correlates with the degree of IL-1 pro-inflammatory stimulation in the TME [366,367]. The underdeveloped pediatric immune system is unable to properly resolve inflammation, and levels of IL-1 are significantly upregulated in pediatric cancer patients contributing to further DNA and tissue damage and metastasis in ES and OS [368,369]. A third critical potent pro-inflammatory cytokine in the TME is IL-6. IL-6 is known to play a role in various physiological processes such as immune function, inflammation, development, and bone metabolism, all of which are affected in pediatric cancer development and metastasis [370]. In adult cancers, acquired oncogenic alterations and age-related immune decline contribute to altered expression of NF-κB, IL-6 (a downstream target of NF-κB), and STAT3 signaling, altering immune recognition and cellular senescence [370,371]. In pediatric cancers, higher levels of IL-6 were detected in patients with poorer prognosis, since IL-6 is known to contribute to accelerated tumor growth [372,373]. Also, TAM production of IL-6 has been shown to support bone metastasis in NB and OS, since IL-6 can stimulate the activity of bone marrow mesenchymal stem cell and bone-resorbing osteoclasts [374,375]. These potent cytokines and others involved with chronic inflammation inhibit the DNA damage response machinery, anti-tumor immunosurveillance, and contribute to malignant genetic and epigenetic changes [48,192].

Given the importance of chronic inflammation in the onset, progression, and treatment response, therapies are being developed to target the effects of inflammation both during and after clinical treatment. For instance, high-risk ALL patients who undergo allogenic hematopoietic stem cell transplant that develop graft-versus-host disease often present with a pro-inflammatory gut microbiome, placing them at risk for chronic adult diseases [376]. Clinical strategies such as nutritional intervention, application of enteral nutrition, targeted antibiotic therapies, and prebiotics are currently under evaluation [377,378]. In OS, the extent of tumor and stromal inflammation correlates with tumor aggressiveness, and therapeutic approaches often include anti-inflammatory drugs [329,379]. Immune checkpoint inhibitors, which function to promote T-cell targeting of cancer cells, have shown considerable effectiveness in treating adult cancers and are currently under evaluation for pediatric malignancies [380,381]. PD-1/PD-L1 blocking drugs, which are effective in adult cancers, have demonstrated mixed effectiveness in pediatric populations due to variations in PD-L1 presentation [326]. However, application of immune checkpoint inhibitors (such as FDA-approved pembrolizumab) have shown promise in the treatment of Hodgkin lymphoma [382,383]. Inhibitors with alternative targets, such as T-cell Ig and mucin domain 3 (TIM-3), indoleamine 2,3-dioxygenase (IDO-1), and B- and T-lymphocyte attenuator (BTLA), are also being explored, though clinical data are scarce [384,385]. Ipilimumab, a therapy targeting cytotoxic T-lymphocyte antigen 4 (CTLA-4), was well tolerated but demonstrated little effectiveness, though it is FDA approved for the treatment of unresectable childhood melanoma [384,386]. Nivolumab is another checkpoint inhibitor FDA approved for use in pediatric patients, where it shows good responses in hypermutated CNS cancers [297,387,388]. Finally, histone deacetylase inhibitors (HDACis), which activate pro-inflammatory transcription factors such as STAT 1/3 and NF-κB, are being investigated for future therapeutic potential to arrest tumor growth and promote cancer cell apoptosis in both in vivo and in vitro models [389,390]. For instance, preclinical studies employing the drug CUDC-907, a dual P13K and HDAC inhibitor, demonstrate a reduced growth rate in NB patients [391]. Also, combination therapy using imipridones drugs alongside other HDACi (such as FDA-approved vorinostat or panobinostat) induced cell death in pre-clinical studies of ES and NB cell lines [392].

The effects of chronic inflammation in the pathogenesis of adult cancers are widely studied. However, recent studies have uncovered significant genetic and epigenetic disparities, resulting in distinct pediatric classifications [34]. Because of this, findings from adult malignancies cannot be directly translated into effective therapeutic strategies in pediatrics. While studies are beginning to explore alterations specific to pediatric TMEs, there is still a scarcity of data regarding the biological significance of the complex interplay between cell populations, the extracellular matrix, and signaling molecules, prompting the need for further studies. In the meantime, insights gleamed into the effects of chronic inflammation in pediatric cancers are being utilized to evaluate novel paradigms for therapies and supportive care.

## Data Availability

The author state that our manuscript is ethically sound and our data acquisition methods meet industry-recognized standards.

## References

[1] Ayub M., Jin H.K., Bae J.-s. (2021). The blood cerebrospinal fluid barrier orchestrates immunosurveillance, immunoprotection, and immunopathology in the central nervous system. BMB Rep..

[2] Moser B., Willimann K. (2004). Chemokines: Role in inflammation and immune surveillance. Ann. Rheum. Dis..

[3] Hassanshahi A., Moradzad M., Ghalamkari S., Fadaei M., Cowin A.J., Hassanshahi M. (2022). Macrophage-mediated inflammation in skin wound healing. Cells.

[4] Headland S.E., Norling L.V. (2015). The resolution of inflammation: Principles and challenges. Seminars in Immunology.

[5] Wolf S.J., Melvin W.J., Gallagher K. (2021). Macrophage-mediated inflammation in diabetic wound repair. Seminars in Cell & Developmental Biology.

[6] Chiang N., Serhan C.N. (2020). Specialized pro-resolving mediator network: An update on production and actions. Essays Biochem..

[7] Hou J., Karin M., Sun B. (2021). Targeting cancer-promoting inflammation—Have anti-inflammatory therapies come of age?. Nat. Rev. Clin. Oncol..

[8] Proto J.D., Doran A.C., Gusarova G., Yurdagul A., Sozen E., Subramanian M., Islam M.N., Rymond C.C., Du J., Hook J. (2018). Regulatory T cells promote macrophage efferocytosis during inflammation resolution. Immunity.

[9] Razi S., Yaghmoorian Khojini J., Kargarijam F., Panahi S., Tahershamsi Z., Tajbakhsh A., Gheibihayat S.M. (2023). Macrophage efferocytosis in health and disease. Cell Biochem. Funct..

[10] Afify S.M., Hassan G., Seno A., Seno M. (2022). Cancer-inducing niche: The force of chronic inflammation. Br. J. Cancer.

[11] Ghorabi S., Esteghamati A., Azam K., Daneshzad E., Sadeghi O., Salari-Moghaddam A., Azadbakht L., Djafarian K. (2020). Association between dietary inflammatory index and components of metabolic syndrome. J. Cardiovasc. Thorac. Res..

[12] Li L.-R., Song J.-L., Liu H.-Q., Chen C. (2023). Metabolic syndrome and thyroid Cancer: Risk, prognosis, and mechanism. Discov. Oncol..

[13] Li Z., Zheng Z., Ruan J., Li Z., Tzeng C.-M. (2016). Chronic inflammation links cancer and Parkinson’s disease. Front. Aging Neurosci..

[14] Libby P. (2021). Inflammation in atherosclerosis—No longer a theory. Clin. Chem..

[15] Rohm T.V., Meier D.T., Olefsky J.M., Donath M.Y. (2022). Inflammation in obesity, diabetes, and related disorders. Immunity.

[16] Alba M.M., Ebright B., Hua B., Slarve I., Zhou Y., Jia Y., Louie S.G., Stiles B.L. (2023). Eicosanoids and other oxylipins in liver injury, inflammation and liver cancer development. Front. Physiol..

[17] Filbin M.G., Tirosh I., Hovestadt V., Shaw M.L., Escalante L.E., Mathewson N.D., Neftel C., Frank N., Pelton K., Hebert C.M. (2018). Developmental and oncogenic programs in H3K27M gliomas dissected by single-cell RNA-seq. Science.

[18] Nigam M., Mishra A.P., Deb V.K., Dimri D.B., Tiwari V., Bungau S.G., Bungau A.F., Radu A.-F. (2023). Evaluation of the association of chronic inflammation and cancer: Insights and implications. Biomed. Pharmacother..

[19] Li Z., Langhans S.A. (2021). In vivo and ex vivo pediatric brain tumor models: An overview. Front. Oncol..

[20] Bray F., Laversanne M., Sung H., Ferlay J., Siegel R.L., Soerjomataram I., Jemal A. (2024). Global cancer statistics 2022: GLOBOCAN estimates of incidence and mortality worldwide for 36 cancers in 185 countries. CA A Cancer J. Clin..

[21] Siegel D.A. (2018). Geographic variation in pediatric cancer incidence—United States, 2003–2014. MMWR Morb. Mortal. Wkly. Rep..

[22] Siegel D.A., King J.B., Lupo P.J., Durbin E.B., Tai E., Mills K., Van Dyne E., Buchanan Lunsford N., Henley S.J., Wilson R.J. (2023). Counts, incidence rates, and trends of pediatric cancer in the United States, 2003-2019. JNCI J. Natl. Cancer Inst..

[23] Siegel D.A., Richardson L.C., Henley S.J., Wilson R.J., Dowling N.F., Weir H.K., Tai E.W., Buchanan Lunsford N. (2020). Pediatric cancer mortality and survival in the United States, 2001–2016. Cancer.

[24] Alduais Y., Zhang H., Fan F., Chen J., Chen B. (2023). Non-small cell lung cancer (NSCLC): A review of risk factors, diagnosis, and treatment. Medicine.

[25] Byrne S., Boyle T., Ahmed M., Lee S.H., Benyamin B., Hyppönen E. (2023). Lifestyle, genetic risk and incidence of cancer: A prospective cohort study of 13 cancer types. Int. J. Epidemiol..

[26] Choi S., Ismail A., Pappas-Gogos G., Boussios S. (2023). HPV and cervical cancer: A review of epidemiology and screening uptake in the UK. Pathogens.

[27] Huber M., Brehm C.U., Gress T.M., Buchholz M., Alashkar Alhamwe B., Pogge von Strandmann E., Slater E.P., Bartsch J.W., Bauer C., Lauth M. (2020). The immune microenvironment in pancreatic cancer. Int. J. Mol. Sci..

[28] Ramachandran D., Dörk T. (2021). Genomic risk factors for cervical cancer. Cancers.

[29] Schmitt M., Greten F.R. (2021). The inflammatory pathogenesis of colorectal cancer. Nat. Rev. Immunol..

[30] Novikov N.M., Zolotaryova S.Y., Gautreau A.M., Denisov E.V. (2021). Mutational drivers of cancer cell migration and invasion. Br. J. Cancer.

[31] Rascio F., Spadaccino F., Rocchetti M.T., Castellano G., Stallone G., Netti G.S., Ranieri E. (2021). The pathogenic role of PI3K/AKT pathway in cancer onset and drug resistance: An updated review. Cancers.

[32] Vargas-Rondón N., Villegas V.E., Rondón-Lagos M. (2017). The role of chromosomal instability in cancer and therapeutic responses. Cancers.

[33] Bertacca I., Pegoraro F., Tondo A., Favre C. (2023). Targeted treatment of solid tumors in pediatric precision oncology. Front. Oncol..

[34] Kattner P., Strobel H., Khoshnevis N., Grunert M., Bartholomae S., Pruss M., Fitzel R., Halatsch M.-E., Schilberg K., Siegelin M.D. (2019). Compare and contrast: Pediatric cancer versus adult malignancies. Cancer Metastasis Rev..

[35] Cunningham R.M., Walton M.A., Carter P.M. (2018). The major causes of death in children and adolescents in the United States. N. Engl. J. Med..

[36] Siegel R.L., Miller K.D., Wagle N.S., Jemal A. (2023). Cancer statistics, 2023. CA A Cancer J. Clin..

[37] Gröbner S.N., Worst B.C., Weischenfeldt J., Buchhalter I., Kleinheinz K., Rudneva V.A., Johann P.D., Balasubramanian G.P., Segura-Wang M., Brabetz S. (2018). The landscape of genomic alterations across childhood cancers. Nature.

[38] Thatikonda V., Islam S.A., Autry R.J., Jones B.C., Gröbner S.N., Warsow G., Hutter B., Huebschmann D., Fröhling S., Kool M. (2023). Comprehensive analysis of mutational signatures reveals distinct patterns and molecular processes across 27 pediatric cancers. Nat. Cancer.

[39] Derks L.L., van Boxtel R. (2023). Stem cell mutations, associated cancer risk, and consequences for regenerative medicine. Cell Stem Cell.

[40] Freire N.H., Jaeger M.d.C., de Farias C.B., Nör C., Souza B.K., Gregianin L., Brunetto A.T., Roesler R. (2023). Targeting the epigenome of cancer stem cells in pediatric nervous system tumors. Mol. Cell. Biochem..

[41] Sanalkumar R., Dong R., Lee L., Xing Y.-H., Iyer S., Letovanec I., La Rosa S., Finzi G., Musolino E., Papait R. (2023). Highly connected 3D chromatin networks established by an oncogenic fusion protein shape tumor cell identity. Sci. Adv..

[42] Babaei G., Aziz S.G.-G., Jaghi N.Z.Z. (2021). EMT, cancer stem cells and autophagy; The three main axes of metastasis. Biomed. Pharmacother..

[43] dos Santos R.P., Roesler R., Gregianin L., Brunetto A.T., da Cunha Jaeger M., Lunardi Brunetto A., de Farias C.B. (2023). Cancer stem cells and chemoresistance in Ewing sarcoma. Curr. Stem Cell Res. Ther..

[44] Zhao J. (2016). Cancer stem cells and chemoresistance: The smartest survives the raid. Pharmacol. Ther..

[45] Huether R., Dong L., Chen X., Wu G., Parker M., Wei L., Ma J., Edmonson M.N., Hedlund E.K., Rusch M.C. (2014). The landscape of somatic mutations in epigenetic regulators across 1,000 paediatric cancer genomes. Nat. Commun..

[46] Cuella-Martin R., Hayward S.B., Fan X., Chen X., Huang J.-W., Taglialatela A., Leuzzi G., Zhao J., Rabadan R., Lu C. (2021). Functional interrogation of DNA damage response variants with base editing screens. Cell.

[47] Groelly F.J., Fawkes M., Dagg R.A., Blackford A.N., Tarsounas M. (2023). Targeting DNA damage response pathways in cancer. Nat. Rev. Cancer.

[48] Klapp V., Álvarez-Abril B., Leuzzi G., Kroemer G., Ciccia A., Galluzzi L. (2023). The DNA damage response and inflammation in cancer. Cancer Discov..

[49] Dersh D., Hollý J., Yewdell J.W. (2021). A few good peptides: MHC class I-based cancer immunosurveillance and immunoevasion. Nat. Rev. Immunol..

[50] Chen M., Linstra R., van Vugt M.A. (2022). Genomic instability, inflammatory signaling and response to cancer immunotherapy. Biochim. Biophys. Acta (BBA)-Rev. Cancer.

[51] Chen X., Agustinus A.S., Li J., DiBona M., Bakhoum S.F. (2024). Chromosomal instability as a driver of cancer progression. Nat. Rev. Genet..

[52] Cinat D., Coppes R.P., Barazzuol L. (2021). DNA damage-induced inflammatory microenvironment and adult stem cell response. Front. Cell Dev. Biol..

[53] Fishbein A., Hammock B.D., Serhan C.N., Panigrahy D. (2021). Carcinogenesis: Failure of resolution of inflammation?. Pharmacol. Ther..

[54] Bakhoum S.F., Ngo B., Laughney A.M., Cavallo J.-A., Murphy C.J., Ly P., Shah P., Sriram R.K., Watkins T.B., Taunk N.K. (2018). Chromosomal instability drives metastasis through a cytosolic DNA response. Nature.

[55] Kumari L., Kumar Y., Bhatia A. (2022). The link between chromosomal instability and immunity in cancer. Handbook of Cancer and Immunology.

[56] Orr B., Compton D.A. (2013). A double-edged sword: How oncogenes and tumor suppressor genes can contribute to chromosomal instability. Front. Oncol..

[57] Sansregret L., Vanhaesebroeck B., Swanton C. (2018). Determinants and clinical implications of chromosomal instability in cancer. Nat. Rev. Clin. Oncol..

[58] Tijhuis A.E., Johnson S.C., McClelland S.E. (2019). The emerging links between chromosomal instability (CIN), metastasis, inflammation and tumour immunity. Mol. Cytogenet..

[59] Jacob K., Albrecht S., Sollier C., Faury D., Sader E., Montpetit A., Serre D., Hauser P., Garami M., Bognár L. (2009). Duplication of 7q34 is specific to juvenile pilocytic astrocytomas and a hallmark of cerebellar and optic pathway tumours. Br. J. Cancer.

[60] Panuciak K., Nowicka E., Mastalerczyk A., Zawitkowska J., Niedźwiecki M., Lejman M. (2023). Overview on aneuploidy in childhood B-cell acute lymphoblastic leukemia. Int. J. Mol. Sci..

[61] Rajan S., Zaccaria S., Cannon M.V., Cam M., Gross A.C., Raphael B.J., Roberts R.D. (2023). Structurally complex osteosarcoma genomes exhibit limited heterogeneity within individual tumors and across evolutionary time. Cancer Res. Commun..

[62] Tonini G.P. (2021). Why Is Aneuploidy Associated with Favorable Outcome in Neuroblastoma?. Biomolecules.

[63] Ceranski A.K., Carreño-Gonzalez M.J., Ehlers A.C., Colombo M.V., Cidre-Aranaz F., Grünewald T.G. (2023). Hypoxia and HIFs in Ewing sarcoma: New perspectives on a multi-facetted relationship. Mol. Cancer.

[64] Liu C.-H., McCord M., Jamshidi P., Sukhanova M., Lu X. (2022). 6. The clinical implications of polyploidy in oligodendrogliomas. Cancer Genet..

[65] Miguel-Fraile P.S., Carrillo-Gijón R., Rodriguez-Peralto J.L., Badiola I.A. (2004). Prognostic significance of DNA ploidy and proliferative index (MIB-1 index) in childhood rhabdomyosarcoma. Am. J. Clin. Pathol..

[66] Pinto M.T., Carcano F.M., Vieira A.G.S., Cabral E.R.M., Lopes L.F. (2021). Molecular biology of pediatric and adult male germ cell tumors. Cancers.

[67] Shah A.T., Azad T.D., Breese M.R., Chabon J.J., Hamilton E.G., Straessler K., Kurtz D.M., Leung S.G., Spillinger A., Liu H.-Y. (2021). A comprehensive circulating tumor DNA assay for detection of translocation and copy-number changes in pediatric sarcomas. Mol. Cancer Ther..

[68] Argani P. (2022). Translocation carcinomas of the kidney. Genes Chromosomes Cancer.

[69] Campregher P., Rosa S., Silveira C., Lima L., de Oliveira J.B., Pelegrino K. (2022). 62. Molecular profile of patients with Acute Myeloid Leukemia at diagnosis. Cancer Genet..

[70] van der Beek J.N., Geller J.I., de Krijger R.R., Graf N., Pritchard-Jones K., Drost J., Verschuur A.C., Murphy D., Ray S., Spreafico F. (2020). Characteristics and outcome of children with renal cell carcinoma: A narrative review. Cancers.

[71] Zou Y.S., Morsberger L., Hardy M., Ghabrial J., Stinnett V., Murry J.B., Long P., Kim A., Pratilas C.A., Llosa N.J. (2023). Complex/cryptic EWSR1:: FLI1/ERG Gene Fusions and 1q Jumping Translocation in Pediatric Ewing Sarcomas. Genes.

[72] Kenny C., O’Meara E., Ulaş M., Hokamp K., O’Sullivan M.J. (2021). Global chromatin changes resulting from single-gene inactivation—The role of SMARCB1 in malignant rhabdoid tumor. Cancers.

[73] Muscat A., Popovski D., Jayasekara W.S.N., Rossello F.J., Ferguson M., Marini K.D., Alamgeer M., Algar E.M., Downie P., Watkins D.N. (2016). Low-dose histone deacetylase inhibitor treatment leads to tumor growth arrest and multi-lineage differentiation of malignant rhabdoid tumors. Clin. Cancer Res..

[74] Navickas S.M., Giles K.A., Brettingham-Moore K.H., Taberlay P.C. (2023). The role of chromatin remodeler SMARCA4/BRG1 in brain cancers: A potential therapeutic target. Oncogene.

[75] Pastorczak A., Krajewska K., Urbanska Z., Szmyd B., Salacinska-Los E., Kobos J., Mlynarski W., Trelinska J. (2021). Ovarian carcinoma in children with constitutional mutation of SMARCA4: Single-family report and literature review. Fam. Cancer.

[76] Byrjalsen A., Hansen T.V., Stoltze U.K., Mehrjouy M.M., Barnkob N.M., Hjalgrim L.L., Mathiasen R., Lautrup C.K., Gregersen P.A., Hasle H. (2020). Nationwide germline whole genome sequencing of 198 consecutive pediatric cancer patients reveals a high incidence of cancer prone syndromes. PLoS Genet..

[77] Newman S., Nakitandwe J., Kesserwan C.A., Azzato E.M., Wheeler D.A., Rusch M., Shurtleff S., Hedges D.J., Hamilton K.V., Foy S.G. (2021). Genomes for kids: The scope of pathogenic mutations in pediatric cancer revealed by comprehensive DNA and RNA sequencing. Cancer Discov..

[78] Ney G.M., McKay L., Koschmann C., Mody R., Li Q. (2020). The emerging role of Ras pathway signaling in pediatric cancer. Cancer Res..

[79] Pilié P.G., Tang C., Mills G.B., Yap T.A. (2019). State-of-the-art strategies for targeting the DNA damage response in cancer. Nat. Rev. Clin. Oncol..

[80] Savary C., Kim A., Lespagnol A., Gandemer V., Pellier I., Andrieu C., Pagès G., Galibert M.-D., Blum Y., de Tayrac M. (2020). Depicting the genetic architecture of pediatric cancers through an integrative gene network approach. Sci. Rep..

[81] Parsons D.W., Roy A., Yang Y., Wang T., Scollon S., Bergstrom K., Kerstein R.A., Gutierrez S., Petersen A.K., Bavle A. (2016). Diagnostic yield of clinical tumor and germline whole-exome sequencing for children with solid tumors. JAMA Oncol..

[82] Zhang J., Walsh M.F., Wu G., Edmonson M.N., Gruber T.A., Easton J., Hedges D., Ma X., Zhou X., Yergeau D.A. (2015). Germline mutations in predisposition genes in pediatric cancer. N. Engl. J. Med..

[83] Baumhoer D., Berthold R., Isfort I., Heinst L., Ameline B., Grünewald I., Thieringer F.M., Rudack C., Wardelmann E., Vieth V. (2022). Recurrent CTNNB1 mutations in craniofacial osteomas. Mod. Pathol..

[84] Capasso M., Montella A., Tirelli M., Maiorino T., Cantalupo S., Iolascon A. (2020). Genetic predisposition to solid pediatric cancers. Front. Oncol..

[85] Feng S., Han L., Yue M., Zhong D., Cao J., Guo Y., Sun Y., Zhang H., Cao Z., Cui X. (2021). Frequency detection of BRAF V600E mutation in a cohort of pediatric langerhans cell histiocytosis patients by next-generation sequencing. Orphanet J. Rare Dis..

[86] He J., Zeng Z., Wang Y., Deng J., Tang X., Liu F., Huang J., Chen H., Liang R., Zan X. (2021). Characterization of novel CTNNB1 mutation in Craniopharyngioma by whole-genome sequencing. Mol. Cancer.

[87] Khaiman C., Techavichit P., Poparn H., Chiengthong K., Lauhasurayotin S., Sosothikul D., Shotelersuk K., Nantavithya C., Jakchairoongruang K., Amornfa J. (2022). KRAS mutation in pediatric intracranial germ cell tumors. Asian Pac. J. Cancer Prev. APJCP.

[88] Nobre L., Zapotocky M., Ramaswamy V., Ryall S., Bennett J., Alderete D., Balaguer Guill J., Baroni L., Bartels U., Bavle A. (2020). Outcomes of BRAF V600E pediatric gliomas treated with targeted BRAF inhibition. JCO Precis. Oncol..

[89] Pondrom M., Bougeard G., Karanian M., Bonneau-Lagacherie J., Boulanger C., Boutroux H., Briandet C., Chevreau C., Corradini N., Coze C. (2020). Rhabdomyosarcoma associated with germline TP53 alteration in children and adolescents: The French experience. Pediatr. Blood Cancer.

[90] Simonin M., Andrieu G.P., Birsen R., Balsat M., Hypolite G., Courtois L., Graux C., Grardel N., Cayuela J.-M., Huguet F. (2023). Prognostic value and oncogenic landscape of TP53 alterations in adult and pediatric T-ALL. Blood.

[91] Talloa D., Triarico S., Agresti P., Mastrangelo S., Attinà G., Romano A., Maurizi P., Ruggiero A. (2022). BRAF and MEK targeted therapies in pediatric central nervous system tumors. Cancers.

[92] Apfelbaum A.A., Wrenn E.D., Lawlor E.R. (2022). The importance of fusion protein activity in Ewing sarcoma and the cell intrinsic and extrinsic factors that regulate it: A review. Front. Oncol..

[93] Bolouri H., Farrar J.E., Triche T., Ries R.E., Lim E.L., Alonzo T.A., Ma Y., Moore R., Mungall A.J., Marra M.A. (2018). The molecular landscape of pediatric acute myeloid leukemia reveals recurrent structural alterations and age-specific mutational interactions. Nat. Med..

[94] Erkizan H.V., Uversky V.N., Toretsky J.A. (2010). Oncogenic partnerships: EWS-FLI1 protein interactions initiate key pathways of Ewing’s sarcoma. Clin. Cancer Res..

[95] Joshi S.K., Qian K., Bisson W.H., Watanabe-Smith K., Huang A., Bottomly D., Traer E., Tyner J.W., McWeeney S.K., Davare M.A. (2020). Discovery and characterization of targetable NTRK point mutations in hematologic neoplasms. Blood J. Am. Soc. Hematol..

[96] Raze T., Lapouble E., Lacour B., Guissou S., Defachelles A.S., Gaspar N., Delattre O., Pierron G., Desandes E. (2023). PAX–FOXO1 fusion status in children and adolescents with alveolar rhabdomyosarcoma: Impact on clinical, pathological, and survival features. Pediatr. Blood Cancer.

[97] Shiba N., Ichikawa H., Taki T., Park M.J., Jo A., Mitani S., Kobayashi T., Shimada A., Sotomatsu M., Arakawa H. (2013). NUP98-NSD1 gene fusion and its related gene expression signature are strongly associated with a poor prognosis in pediatric acute myeloid leukemia. Genes Chromosomes Cancer.

[98] Sweet-Cordero E.A., Biegel J.A. (2019). The genomic landscape of pediatric cancers: Implications for diagnosis and treatment. Science.

[99] Cavalli F.M., Remke M., Rampasek L., Peacock J., Shih D.J., Luu B., Garzia L., Torchia J., Nor C., Morrissy A.S. (2017). Intertumoral heterogeneity within medulloblastoma subgroups. Cancer Cell.

[100] Lazow M.A., Hoffman L., Schafer A., Osorio D.S., Boué D.R., Rush S., Wright E., Lane A., DeWire-Schottmiller M.D., Smolarek T. (2020). Characterizing temporal genomic heterogeneity in pediatric low-grade gliomas. Acta Neuropathol. Commun..

[101] Liu Z., Chen S.S., Clarke S., Veschi V., Thiele C.J. (2021). Targeting MYCN in pediatric and adult cancers. Front. Oncol..

[102] Ostrom Q.T., Cioffi G., Gittleman H., Patil N., Waite K., Kruchko C., Barnholtz-Sloan J.S. (2019). CBTRUS statistical report: Primary brain and other central nervous system tumors diagnosed in the United States in 2012–2016. Neuro-Oncology.

[103] Plant-Fox A.S., O’Halloran K., Goldman S. (2021). Pediatric brain tumors: The era of molecular diagnostics, targeted and immune-based therapeutics, and a focus on long term neurologic sequelae. Curr. Probl. Cancer.

[104] Aubin R.G., Troisi E.C., Montelongo J., Alghalith A.N., Nasrallah M.P., Santi M., Camara P.G. (2022). Pro-inflammatory cytokines mediate the epithelial-to-mesenchymal-like transition of pediatric posterior fossa ependymoma. Nat. Commun..

[105] Kartikasari A.E., Huertas C.S., Mitchell A., Plebanski M. (2021). Tumor-induced inflammatory cytokines and the emerging diagnostic devices for cancer detection and prognosis. Front. Oncol..

[106] Panina S.B., Baran N., Brasil da Costa F.H., Konopleva M., Kirienko N.V. (2019). A mechanism for increased sensitivity of acute myeloid leukemia to mitotoxic drugs. Cell Death Dis..

[107] Perrone S., Lotti F., Geronzi U., Guidoni E., Longini M., Buonocore G. (2016). Oxidative Stress in Cancer-Prone Genetic Diseases in Pediatric Age: The Role of Mitochondrial Dysfunction. Oxidative Med. Cell. Longev..

[108] Raber M., Wu J., Donnella H., Knouse P., Pise M., Munsell M., Liu D., Chandra J. (2019). Cellular oxidative stress in pediatric leukemia and lymphoma patients undergoing treatment is associated with protein consumption. Nutrients.

[109] Saleh E.A.M., Al-Dolaimy F., Baymakov S., Ullah M.I., Khlewee I.H., Bisht Y.S., Alsaalamy A.H. (2023). Oxidative stress affects the beginning of the growth of cancer cells through a variety of routes. Pathol.-Res. Pract..

[110] Fuchs Q., Pierrevelcin M., Messe M., Lhermitte B., Blandin A.-F., Papin C., Coca A., Dontenwill M., Entz-Werlé N. (2020). Hypoxia inducible factors’ signaling in pediatric high-grade gliomas: Role, modelization and innovative targeted approaches. Cancers.

[111] Pierrevelcin M., Fuchs Q., Lhermitte B., Messé M., Guérin E., Weingertner N., Martin S., Lelong-Rebel I., Nazon C., Dontenwill M. (2020). Focus on hypoxia-related pathways in pediatric osteosarcomas and their druggability. Cells.

[112] Wang M.Y., Liow P., Guzman M.I.T., Qi J. (2022). Exploring methods of targeting histone methyltransferases and their applications in cancer therapeutics. ACS Chem. Biol..

[113] Wang Y., Qi H., Liu Y., Duan C., Liu X., Xia T., Chen D., Piao H.-l., Liu H.-X. (2021). The double-edged roles of ROS in cancer prevention and therapy. Theranostics.

[114] Ball H.C., Alejo A.L., Samson T.K., Alejo A.M., Safadi F.F. (2022). Epigenetic regulation of chondrocytes and subchondral bone in osteoarthritis. Life.

[115] Benincasa G., DeMeo D.L., Glass K., Silverman E.K., Napoli C. (2021). Epigenetics and pulmonary diseases in the horizon of precision medicine: A review. Eur. Respir. J..

[116] Lee C., Kim J.K. (2021). Chromatin regulators in retinoblastoma: Biological roles and therapeutic applications. J. Cell. Physiol..

[117] Zhang L., Lu Q., Chang C. (2020). Epigenetics in health and disease. Epigenetics Allergy Autoimmun..

[118] Morel D., Jeffery D., Aspeslagh S., Almouzni G., Postel-Vinay S. (2020). Combining epigenetic drugs with other therapies for solid tumours—Past lessons and future promise. Nat. Rev. Clin. Oncol..

[119] Mosaab A., El-Ayadi M., Khorshed E.N., Amer N., Refaat A., El-Beltagy M., Hassan Z., Soror S.H., Zaghloul M.S., El-Naggar S. (2020). Histone H3K27M mutation overrides histological grading in pediatric gliomas. Sci. Rep..

[120] Niu J., Peng D., Liu L. (2022). Drug resistance mechanisms of acute myeloid leukemia stem cells. Front. Oncol..

[121] Rakotomalala A., Bailleul Q., Savary C., Arcicasa M., Hamadou M., Huchedé P., Hochart A., Restouin A., Castellano R., Collette Y. (2021). H3. 3K27M mutation controls cell growth and resistance to therapies in pediatric glioma cell lines. Cancers.

[122] Johann P.-D. (2020). Invited Review: Dysregulation of chromatin remodellers in paediatric brain tumours–SMARCB1 and beyond. Neuropathol. Appl. Neurobiol..

[123] Pathania A.S. (2023). Crosstalk between Noncoding RNAs and the Epigenetics Machinery in Pediatric Tumors and Their Microenvironment. Cancers.

[124] Perla A., Fratini L., Cardoso P.S., Nör C., Brunetto A.T., Brunetto A.L., de Farias C.B., Jaeger M., Roesler R. (2020). Histone deacetylase inhibitors in pediatric brain cancers: Biological activities and therapeutic potential. Front. Cell Dev. Biol..

[125] French R., Pauklin S. (2021). Epigenetic regulation of cancer stem cell formation and maintenance. Int. J. Cancer.

[126] Novak D., Hüser L., Elton J.J., Umansky V., Altevogt P., Utikal J. (2020). SOX2 in development and cancer biology. Seminars in Cancer Biology.

[127] Wu S., Tan Y., Li F., Han Y., Zhang S., Lin X. (2024). CD44: A cancer stem cell marker and therapeutic target in leukemia treatment. Front. Immunol..

[128] Celarain N., Tomas-Roig J. (2020). Aberrant DNA methylation profile exacerbates inflammation and neurodegeneration in multiple sclerosis patients. J. Neuroinflammation.

[129] Gasparetto M., Payne F., Nayak K., Kraiczy J., Glemas C., Philip-McKenzie Y., Ross A., Edgar R.D., Zerbino D.R., Salvestrini C. (2021). Transcription and DNA methylation patterns of blood-derived CD8+ T cells are associated with age and inflammatory bowel disease but do not predict prognosis. Gastroenterology.

[130] Ligthart S., Marzi C., Aslibekyan S., Mendelson M.M., Conneely K.N., Tanaka T., Colicino E., Waite L.L., Joehanes R., Guan W. (2016). DNA methylation signatures of chronic low-grade inflammation are associated with complex diseases. Genome Biol..

[131] Myte R., Sundkvist A., Van Guelpen B., Harlid S. (2019). Circulating levels of inflammatory markers and DNA methylation, an analysis of repeated samples from a population based cohort. Epigenetics.

[132] Stenvinkel P., Karimi M., Johansson S., Axelsson J., Suliman M., Lindholm B., Heimbürger O., Barany P., Alvestrand A., Nordfors L. (2007). Impact of inflammation on epigenetic DNA methylation–a novel risk factor for cardiovascular disease?. J. Intern. Med..

[133] Forno E., Wang T., Qi C., Yan Q., Xu C.-J., Boutaoui N., Han Y.-Y., Weeks D.E., Jiang Y., Rosser F. (2019). DNA methylation in nasal epithelium, atopy, and atopic asthma in children: A genome-wide study. Lancet Respir. Med..

[134] Howell K.J., Kraiczy J., Nayak K.M., Gasparetto M., Ross A., Lee C., Mak T.N., Koo B.-K., Kumar N., Lawley T. (2018). DNA methylation and transcription patterns in intestinal epithelial cells from pediatric patients with inflammatory bowel diseases differentiate disease subtypes and associate with outcome. Gastroenterology.

[135] Somineni H.K., Venkateswaran S., Kilaru V., Marigorta U.M., Mo A., Okou D.T., Kellermayer R., Mondal K., Cobb D., Walters T.D. (2019). Blood-derived DNA methylation signatures of Crohn’s disease and severity of intestinal inflammation. Gastroenterology.

[136] Yan Q., Forno E., Cardenas A., Qi C., Han Y.Y., Acosta-Pérez E., Kim S., Zhang R., Boutaoui N., Canino G. (2021). Exposure to violence, chronic stress, nasal DNA methylation, and atopic asthma in children. Pediatr. Pulmonol..

[137] Gujar H., Weisenberger D.J., Liang G. (2019). The roles of human DNA methyltransferases and their isoforms in shaping the epigenome. Genes.

[138] Loaeza-Loaeza J., Beltran A.S., Hernández-Sotelo D. (2020). DNMTs and impact of CpG content, transcription factors, consensus motifs, lncRNAs, and histone marks on DNA methylation. Genes.

[139] Chen Z., Zhang Y. (2020). Role of mammalian DNA methyltransferases in development. Annu. Rev. Biochem..

[140] Blackledge N.P., Klose R. (2011). CpG island chromatin: A platform for gene regulation. Epigenetics.

[141] Angeloni A., Bogdanovic O. (2021). Sequence determinants, function, and evolution of CpG islands. Biochem. Soc. Trans..

[142] Deaton A.M., Bird A. (2011). CpG islands and the regulation of transcription. Genes Dev..

[143] Kang G.H., Lee S., Kim J.-S., Jung H.-Y. (2003). Profile of aberrant CpG island methylation along the multistep pathway of gastric carcinogenesis. Lab. Investig..

[144] Li Y., Liu S., Wang H., Mai H., Yuan X., Li C., Chen X., Wen F. (2017). Methylation level of CpG islands in GGH gene promoter in pediatric acute leukemia. PLoS ONE.

[145] Lietz C.E., Newman E.T., Kelly A.D., Xiang D.H., Zhang Z., Luscko C.A., Lozano-Calderon S.A., Ebb D.H., Raskin K.A., Cote G.M. (2022). Genome-wide DNA methylation patterns reveal clinically relevant predictive and prognostic subtypes in human osteosarcoma. Commun. Biol..

[146] Cristalli C., Scotlandi K. (2024). Targeting DNA Methylation Machinery in Pediatric Solid Tumors. Cells.

[147] Esteller M., Herman J.G. (2004). Generating mutations but providing chemosensitivity: The role of O6-methylguanine DNA methyltransferase in human cancer. Oncogene.

[148] Bueno A.C., da Silva R.M., Stecchini M.F., Marrero-Gutiérrez J., e Silva D.C.d.A., Cardinalli I., Scrideli C.A., Junqueira T., Molina C.A., Ramalho F.S. (2022). DNA methylation is a comprehensive marker for pediatric adrenocortical tumors. Endocr.-Relat. Cancer.

[149] Krali O., Palle J., Bäcklin C.L., Abrahamsson J., Norén-Nyström U., Hasle H., Jahnukainen K., Jónsson Ó.G., Hovland R., Lausen B. (2021). DNA methylation signatures predict cytogenetic subtype and outcome in pediatric acute myeloid leukemia (AML). Genes.

[150] Lalchungnunga H., Hao W., Maris J.M., Asgharzadeh S., Henrich K.-O., Westermann F., Tweddle D.A., Schwalbe E.C., Strathdee G. (2022). Genome wide DNA methylation analysis identifies novel molecular subgroups and predicts survival in neuroblastoma. Br. J. Cancer.

[151] Saint Fleur-Lominy S., Evensen N.A., Bhatla T., Sethia G., Narang S., Choi J.H., Ma X., Yang J.J., Kelly S., Raetz E. (2020). Evolution of the epigenetic landscape in childhood B acute lymphoblastic leukemia and its role in drug resistance. Cancer Res..

[152] Vargas A.C., Gray L.-A., White C.L., Maclean F.M., Grimison P., Ardakani N.M., Bonar F., Algar E.M., Cheah A.L., Russell P. (2021). Genome wide methylation profiling of selected matched soft tissue sarcomas identifies methylation changes in metastatic and recurrent disease. Sci. Rep..

[153] Aberuyi N., Rahgozar S., Ghodousi E.S., Ghaedi K. (2020). Drug resistance biomarkers and their clinical applications in childhood acute lymphoblastic leukemia. Front. Oncol..

[154] Hu C., Liu X., Zeng Y., Liu J., Wu F. (2021). DNA methyltransferase inhibitors combination therapy for the treatment of solid tumor: Mechanism and clinical application. Clin. Epigenetics.

[155] Romero-Garcia S., Prado-Garcia H., Carlos-Reyes A. (2020). Role of DNA methylation in the resistance to therapy in solid tumors. Front. Oncol..

[156] Fortuny A., Chansard A., Caron P., Chevallier O., Leroy O., Renaud O., Polo S.E. (2021). Imaging the response to DNA damage in heterochromatin domains reveals core principles of heterochromatin maintenance. Nat. Commun..

[157] Martire S., Banaszynski L.A. (2020). The roles of histone variants in fine-tuning chromatin organization and function. Nat. Rev. Mol. Cell Biol..

[158] Jenuwein T., Allis C.D. (2001). Translating the histone code. Science.

[159] Mohammad F., Helin K. (2017). Oncohistones: Drivers of pediatric cancers. Genes Dev..

[160] Zandian M., Gonzalez Salguero N., Shannon M.D., Purusottam R.N., Theint T., Poirier M.G., Jaroniec C.P. (2021). Conformational dynamics of histone H3 tails in chromatin. J. Phys. Chem. Lett..

[161] Zhang Y., Sun Z., Jia J., Du T., Zhang N., Tang Y., Fang Y., Fang D. (2021). Overview of histone modification. Histone Mutat. Cancer.

[162] Gulati R., Fleifil Y., Jennings K., Bondoc A., Tiao G., Geller J., Timchenko L., Timchenko N. (2024). Inhibition of histone deacetylase activity increases cisplatin efficacy to eliminate metastatic cells in pediatric liver cancers. Cancers.

[163] Neganova M.E., Klochkov S.G., Aleksandrova Y.R., Aliev G. (2022). Histone modifications in epigenetic regulation of cancer: Perspectives and achieved progress. Seminars in Cancer Biology.

[164] Rugo H.S., Jacobs I., Sharma S., Scappaticci F., Paul T.A., Jensen-Pergakes K., Malouf G.G. (2020). The promise for histone methyltransferase inhibitors for epigenetic therapy in clinical oncology: A narrative review. Adv. Ther..

[165] Di Lorenzo A., Bedford M.T. (2011). Histone arginine methylation. FEBS Lett..

[166] Akiyama T., Wakabayashi S., Soma A., Sato S., Nakatake Y., Oda M., Murakami M., Sakota M., Chikazawa-Nohtomi N., Ko S.B. (2016). Transient ectopic expression of the histone demethylase JMJD3 accelerates the differentiation of human pluripotent stem cells. Development.

[167] Ding Y., Yao Y., Gong X., Zhuo Q., Chen J., Tian M., Farzaneh M. (2021). JMJD3: A critical epigenetic regulator in stem cell fate. Cell Commun. Signal..

[168] Shan Y., Zhang Y., Zhao Y., Wang T., Zhang J., Yao J., Ma N., Liang Z., Huang W., Huang K. (2020). JMJD3 and UTX determine fidelity and lineage specification of human neural progenitor cells. Nat. Commun..

[169] Bayarsaihan D. (2011). Epigenetic mechanisms in inflammation. J. Dent. Res..

[170] De Santa F., Totaro M.G., Prosperini E., Notarbartolo S., Testa G., Natoli G. (2007). The histone H3 lysine-27 demethylase Jmjd3 links inflammation to inhibition of polycomb-mediated gene silencing. Cell.

[171] An S., Camarillo J.M., Huang T.Y.-T., Li D., Morris J.A., Zoltek M.A., Qi J., Behbahani M., Kambhampati M., Kelleher N.L. (2020). Histone tail analysis reveals H3K36me2 and H4K16ac as epigenetic signatures of diffuse intrinsic pontine glioma. J. Exp. Clin. Cancer Res..

[172] Badodi S., Marino S. (2022). Epigenetic mechanisms in paediatric brain tumours: Regulators lose control. Biochem. Soc. Trans..

[173] Klonou A., Korkolopoulou P., Gargalionis A.N., Kanakoglou D.S., Katifelis H., Gazouli M., Chlamydas S., Mitsios A., Kalamatianos T., Stranjalis G. (2021). Histone mark profiling in pediatric astrocytomas reveals prognostic significance of H3K9 trimethylation and histone methyltransferase SUV39H1. Neurotherapeutics.

[174] Klonou A., Korkolopoulou P., Giannopoulou A.-I., Kanakoglou D.S., Pampalou A., Gargalionis A.N., Sarantis P., Mitsios A., Sgouros S., Papavassiliou A.G. (2023). Histone H3K9 methyltransferase SETDB1 overexpression correlates with pediatric high-grade gliomas progression and prognosis. J. Mol. Med..

[175] Sepsa A., Levidou G., Gargalionis A., Adamopoulos C., Spyropoulou A., Dalagiorgou G., Thymara I., Boviatsis E., Themistocleous M.S., Petraki K. (2015). Emerging role of linker histone variant H1x as a biomarker with prognostic value in astrocytic gliomas. A multivariate analysis including trimethylation of H3K9 and H4K20. PLoS ONE.

[176] Zhou M., Li Y., Lin S., Chen Y., Qian Y., Zhao Z., Fan H. (2019). H3K9me3, H3K36me3, and H4K20me3 expression correlates with patient outcome in esophageal squamous cell carcinoma as epigenetic markers. Dig. Dis. Sci..

[177] Agredo A., Kasinski A.L. (2023). Histone 4 lysine 20 tri-methylation: A key epigenetic regulator in chromatin structure and disease. Front. Genet..

[178] Vinci M., Burford A., Molinari V., Kessler K., Popov S., Clarke M., Taylor K.R., Pemberton H.N., Lord C.J., Gutteridge A. (2018). Functional diversity and cooperativity between subclonal populations of pediatric glioblastoma and diffuse intrinsic pontine glioma cells. Nat. Med..

[179] Xu H., Wen Y., Jin R., Chen H. (2022). Epigenetic modifications and targeted therapy in pediatric acute myeloid leukemia. Front. Pediatr..

[180] Dubuc A.M., Remke M., Korshunov A., Northcott P.A., Zhan S.H., Mendez-Lago M., Kool M., Jones D.T., Unterberger A., Morrissy A.S. (2013). Aberrant patterns of H3K4 and H3K27 histone lysine methylation occur across subgroups in medulloblastoma. Acta Neuropathol..

[181] Lewis R., Li Y.D., Hoffman L., Hashizume R., Gravohac G., Rice G., Wadhwani N.R., Jie C., Pundy T., Mania-Farnell B. (2019). Global reduction of H3K4me3 improves chemotherapeutic efficacy for pediatric ependymomas. Neoplasia.

[182] Roussel M.F., Stripay J.L. (2018). Epigenetic drivers in pediatric medulloblastoma. Cerebellum.

[183] Bressan R.B., Southgate B., Ferguson K.M., Blin C., Grant V., Alfazema N., Wills J.C., Marques-Torrejon M.A., Morrison G.M., Ashmore J. (2021). Regional identity of human neural stem cells determines oncogenic responses to histone H3. 3 mutants. Cell Stem Cell.

[184] Huang R., Wu Y., Zou Z. (2022). Combining EZH2 inhibitors with other therapies for solid tumors: More choices for better effects. Epigenomics.

[185] Huang T.Y.-T., Piunti A., Qi J., Morgan M., Bartom E., Shilatifard A., Saratsis A.M. (2020). Effects of H3. 3G34V mutation on genomic H3K36 and H3K27 methylation patterns in isogenic pediatric glioma cells. Acta Neuropathol. Commun..

[186] Shi L., Shi J., Shi X., Li W., Wen H. (2018). Histone H3. 3 G34 mutations alter histone H3K36 and H3K27 methylation in cis. J. Mol. Biol..

[187] Jain S.U., Khazaei S., Marchione D.M., Lundgren S.M., Wang X., Weinberg D.N., Deshmukh S., Juretic N., Lu C., Allis C.D. (2020). Histone H3. 3 G34 mutations promote aberrant PRC2 activity and drive tumor progression. Proc. Natl. Acad. Sci. USA.

[188] Moritz L.E., Trievel R.C. (2018). Structure, mechanism, and regulation of polycomb-repressive complex 2. J. Biol. Chem..

[189] Parreno V., Martinez A.-M., Cavalli G. (2022). Mechanisms of Polycomb group protein function in cancer. Cell Res..

[190] Becker J.S., Nicetto D., Zaret K.S. (2016). H3K9me3-dependent heterochromatin: Barrier to cell fate changes. Trends Genet..

[191] Béguelin W., Popovic R., Teater M., Jiang Y., Bunting K.L., Rosen M., Shen H., Yang S.N., Wang L., Ezponda T. (2013). EZH2 is required for germinal center formation and somatic EZH2 mutations promote lymphoid transformation. Cancer Cell.

[192] Das P., Taube J.H. (2020). Regulating methylation at H3K27: A trick or treat for cancer cell plasticity. Cancers.

[193] Lund K., Adams P., Copland M. (2014). EZH2 in normal and malignant hematopoiesis. Leukemia.

[194] Nacev B.A., Jones K.B., Intlekofer A.M., Yu J.S., Allis C.D., Tap W.D., Ladanyi M., Nielsen T.O. (2020). The epigenomics of sarcoma. Nat. Rev. Cancer.

[195] Goldstein M. (2023). Targeting H3K27me3 loss in pediatric brain tumors-a perspective on epigenetically guided cancer therapy. Oncotarget.

[196] Grunstein M. (1997). Histone acetylation in chromatin structure and transcription. Nature.

[197] Cress W.D., Seto E. (2000). Histone deacetylases, transcriptional control, and cancer. J. Cell. Physiol..

[198] Gallinari P., Marco S.D., Jones P., Pallaoro M., Steinkühler C. (2007). HDACs, histone deacetylation and gene transcription: From molecular biology to cancer therapeutics. Cell Res..

[199] Zhao S., Zhang X., Li H. (2018). Beyond histone acetylation—Writing and erasing histone acylations. Curr. Opin. Struct. Biol..

[200] Bhattacharya D., Pomeroy S.L., Pomeranz Krummel D.A., Sengupta S. (2020). Epigenetics and survivorship in pediatric brain tumor patients. J. Neuro-Oncol..

[201] Brassesco M.S., Roberto G.M., Delsin L.E., Baldissera G.C., Medeiros M., Umezawa K., Tone L.G. (2023). A foretaste for pediatric glioblastoma therapy: Targeting the NF-kB pathway with DHMEQ. Child’s Nerv. Syst..

[202] Kumar V., Palermo R., Talora C., Campese A.F., Checquolo S., Bellavia D., Tottone L., Testa G., Miele E., Indraccolo S. (2014). Notch and NF-kB signaling pathways regulate miR-223/FBXW7 axis in T-cell acute lymphoblastic leukemia. Leukemia.

[203] Medeiros M., Candido M.F., Valera E.T., Brassesco M.S. (2021). The multifaceted NF-kB: Are there still prospects of its inhibition for clinical intervention in pediatric central nervous system tumors?. Cell. Mol. Life Sci..

[204] Zhang S.-Y., Zhang L.-Y., Wen R., Yang N., Zhang T.-N. (2024). Histone deacetylases and their inhibitors in inflammatory diseases. Biomed. Pharmacother..

[205] Ding L.-W., Sun Q.-Y., Tan K.-T., Chien W., Thippeswamy A.M., Eng Juh Yeoh A., Kawamata N., Nagata Y., Xiao J.-F., Loh X.-Y. (2017). Mutational landscape of pediatric acute lymphoblastic leukemia. Cancer Res..

[206] Ding W., Wang D., Cai M., Yan Y., Liu S., Liu X., Luo A., Deng D., Liu X., Jiang H. (2023). PIWIL1 gene polymorphism and pediatric acute lymphoblastic leukemia relapse susceptibility among Chinese children: A five-center case–control study. Front. Oncol..

[207] Gao Y.-y., Ling Z.-y., Zhu Y.-R., Shi C., Wang Y., Zhang X.-y., Zhang Z.-q., Jiang Q., Chen M.-B., Yang S. (2021). The histone acetyltransferase HBO1 functions as a novel oncogenic gene in osteosarcoma. Theranostics.

[208] Lamble A.J., Gerbing R.B., Smith J.L., Ries R.E., Kolb E.A., Alonzo T.A., Meshinchi S. (2021). Crebbp alterations are associated with a poor prognosis in de novo AML. Blood.

[209] Milde T., Oehme I., Korshunov A., Kopp-Schneider A., Remke M., Northcott P., Deubzer H.E., Lodrini M., Taylor M.D., Von Deimling A. (2010). HDAC5 and HDAC9 in medulloblastoma: Novel markers for risk stratification and role in tumor cell growth. Clin. Cancer Res..

[210] Rivas M., Johnston II M.E., Gulati R., Kumbaji M., Aguiar T.F.M., Timchenko L., Krepischi A., Shin S., Bondoc A., Tiao G. (2021). HDAC1-dependent repression of markers of hepatocytes and P21 is involved in development of pediatric liver cancer. Cell. Mol. Gastroenterol. Hepatol..

[211] Shamloo B., Usluer S. (2019). p21 in cancer research. Cancers.

[212] Charlab R., Racz R. (2023). The expanding universe of NUTM1 fusions in pediatric cancer. Clin. Transl. Sci..

[213] Kotekar A., Singh A.K., Devaiah B.N. (2023). BRD4 and MYC: Power couple in transcription and disease. FEBS J..

[214] Iizuka M., Takahashi Y., Mizzen C.A., Cook R.G., Fujita M., Allis C.D., Frierson H.F., Fukusato T., Smith M.M. (2009). Histone acetyltransferase Hbo1: Catalytic activity, cellular abundance, and links to primary cancers. Gene.

[215] Roussel M.F., Robinson G.W. (2013). Role of MYC in Medulloblastoma. Cold Spring Harb. Perspect. Med..

[216] Schwalbe E.C., Lindsey J.C., Danilenko M., Hill R.M., Crosier S., Ryan S.L., Williamson D., Castle J., Hicks D., Kool M. (2024). Molecular and clinical heterogeneity within MYC-family amplified medulloblastoma is associated with survival outcomes: A multicenter cohort study. Neuro-Oncology.

[217] Ecker J., Thatikonda V., Sigismondo G., Selt F., Valinciute G., Oehme I., Müller C., Buhl J.L., Ridinger J., Usta D. (2021). Reduced chromatin binding of MYC is a key effect of HDAC inhibition in MYC amplified medulloblastoma. Neuro-Oncology.

[218] Shofuda T., Kanemura Y. (2021). HDACs and MYC in Medulloblastoma: How Do HDAC Inhibitors Control MYC-Amplified Tumors?. Neuro-oncology.

[219] Vlasevska S., Garcia-Ibanez L., Duval R., Holmes A.B., Jahan R., Cai B., Kim A., Mo T., Basso K., Soni R.K. (2023). KMT2D acetylation by CREBBP reveals a cooperative functional interaction at enhancers in normal and malignant germinal center B cells. Proc. Natl. Acad. Sci. USA.

[220] Zhu Y., Wang Z., Li Y., Peng H., Liu J., Zhang J., Xiao X. (2023). The role of CREBBP/EP300 and its therapeutic implications in hematological malignancies. Cancers.

[221] Khodarev N.N., Roizman B., Weichselbaum R.R. (2012). Molecular pathways: Interferon/stat1 pathway: Role in the tumor resistance to genotoxic stress and aggressive growth. Clin. Cancer Res..

[222] Xiong F., Wang D., Xiong W., Wang X., Huang W.-h., Wu G.-h., Liu W.-z., Wang Q., Chen J.-s., Kuai Y.-y. (2024). Unveiling the role of HP1α-HDAC1-STAT1 axis as a therapeutic target for HP1α-positive intrahepatic cholangiocarcinoma. J. Exp. Clin. Cancer Res..

[223] Rong D., Sun G., Wu F., Cheng Y., Sun G., Jiang W., Li X., Zhong Y., Wu L., Zhang C. (2021). Epigenetics: Roles and therapeutic implications of non-coding RNA modifications in human cancers. Mol. Ther.-Nucleic Acids.

[224] Wei J.-W., Huang K., Yang C., Kang C.-S. (2017). Non-coding RNAs as regulators in epigenetics. Oncol. Rep..

[225] Ashrafizadeh M., Zarrabi A., Mostafavi E., Aref A.R., Sethi G., Wang L., Tergaonkar V. (2022). Non-coding RNA-based regulation of inflammation. Seminars in Immunology.

[226] Ginckels P., Holvoet P. (2022). Focus: The science of stress: Oxidative stress and inflammation in cardiovascular diseases and cancer: Role of non-coding RNAs. Yale J. Biol. Med..

[227] Bure I.V., Nemtsova M.V., Kuznetsova E.B. (2022). Histone modifications and non-coding RNAs: Mutual epigenetic regulation and role in pathogenesis. Int. J. Mol. Sci..

[228] Farooqi A.A., Fayyaz S., Poltronieri P., Calin G., Mallardo M. (2022). Epigenetic deregulation in cancer: Enzyme players and non-coding RNAs. Seminars in Cancer Biology.

[229] Morselli M., Dieci G. (2022). Epigenetic regulation of human non-coding RNA gene transcription. Biochem. Soc. Trans..

[230] Della Bella E., Koch J., Baerenfaller K. (2022). Translation and emerging functions of non-coding RNAs in inflammation and immunity. Allergy.

[231] Green J., Ansari M., Ball H., Haqqi T. (2020). tRNA-derived fragments (tRFs) regulate post-transcriptional gene expression via AGO-dependent mechanism in IL-1β stimulated chondrocytes. Osteoarthr. Cartil..

[232] Mattick J.S., Amaral P.P., Carninci P., Carpenter S., Chang H.Y., Chen L.-L., Chen R., Dean C., Dinger M.E., Fitzgerald K.A. (2023). Long non-coding RNAs: Definitions, functions, challenges and recommendations. Nat. Rev. Mol. Cell Biol..

[233] Tsagakis I., Douka K., Birds I., Aspden J.L. (2020). Long non-coding RNAs in development and disease: Conservation to mechanisms. J. Pathol..

[234] Eldesouki S., Samara K.A., Qadri R., Obaideen A.A., Otour A.H., Habbal O., Ahmed S.B. (2022). XIST in brain cancer. Clin. Chim. Acta.

[235] Nesterova T.B., Wei G., Coker H., Pintacuda G., Bowness J.S., Zhang T., Almeida M., Bloechl B., Moindrot B., Carter E.J. (2019). Systematic allelic analysis defines the interplay of key pathways in X chromosome inactivation. Nat. Commun..

[236] Sahakyan A., Yang Y., Plath K. (2018). The role of Xist in X-chromosome dosage compensation. Trends Cell Biol..

[237] Loh C.-Y., Chai J.Y., Tang T.F., Wong W.F., Sethi G., Shanmugam M.K., Chong P.P., Looi C.Y. (2019). The E-cadherin and N-cadherin switch in epithelial-to-mesenchymal transition: Signaling, therapeutic implications, and challenges. Cells.

[238] Salehi F., Agur A., Scheithauer B.W., Kovacs K., Lloyd R.V., Cusimano M. (2010). Biomarkers of pituitary neoplasms: A review (Part II). Neurosurgery.

[239] Yang H., Zhang X., Zhao Y., Sun G., Zhang J., Gao Y., Liu Q., Zhang W., Zhu H. (2020). Downregulation of lncRNA XIST represses tumor growth and boosts radiosensitivity of neuroblastoma via modulation of the miR-375/L1CAM Axis. Neurochem. Res..

[240] Akpa M.M., Iglesias D., Chu L., Thiébaut A., Jentoft I., Hammond L., Torban E., Goodyer P.R. (2016). Wilms tumor suppressor, WT1, cooperates with microRNA-26a and microRNA-101 to suppress translation of the polycomb protein, EZH2, in mesenchymal stem cells. J. Biol. Chem..

[241] Illarregi U., Telleria J., Bilbao-Aldaiturriaga N., Lopez-Lopez E., Ballesteros J., Martin-Guerrero I., Gutierrez-Camino A. (2022). lncRNA deregulation in childhood acute lymphoblastic leukemia: A systematic review. Int. J. Oncol..

[242] Liu F., Xiong Q.-W., Wang J.-H., Peng W.-X. (2023). Roles of lncRNAs in childhood cancer: Current landscape and future perspectives. Front. Oncol..

[243] Meng X., Zhang Y., Hu Y., Zhong J., Jiang C., Zhang H. (2021). LncRNA CCAT1 sponges miR-218-5p to promote EMT, cellular migration and invasion of retinoblastoma by targeting MTF2. Cell. Signal..

[244] Rea J., Carissimo A., Trisciuoglio D., Illi B., Picard D., Remke M., Laneve P., Caffarelli E. (2021). Identification and functional characterization of novel MYC-regulated long noncoding RNAs in group 3 medulloblastoma. Cancers.

[245] Carvalho de Oliveira J., Molinari Roberto G., Baroni M., Bezerra Salomão K., Alejandra Pezuk J., Sol Brassesco M. (2018). MiRNA dysregulation in childhood hematological cancer. Int. J. Mol. Sci..

[246] Deffenbacher K.E., Iqbal J., Sanger W., Shen Y., Lachel C., Liu Z., Liu Y., Lim M.S., Perkins S.L., Fu K. (2012). Molecular distinctions between pediatric and adult mature B-cell non-Hodgkin lymphomas identified through genomic profiling. Blood J. Am. Soc. Hematol..

[247] Galardi A., Colletti M., Di Paolo V., Vitullo P., Antonetti L., Russo I., Di Giannatale A. (2019). Exosomal MiRNAs in pediatric cancers. Int. J. Mol. Sci..

[248] Abedalthagafi M., Mobark N., Al-Rashed M., AlHarbi M. (2021). Epigenomics and immunotherapeutic advances in pediatric brain tumors. NPJ Precis. Oncol..

[249] Gruszka R., Zakrzewski J., Nowosławska E., Grajkowska W., Zakrzewska M. (2024). Identification and validation of miRNA-target genes network in pediatric brain tumors. Sci. Rep..

[250] Hovestadt V., Smith K.S., Bihannic L., Filbin M.G., Shaw M.L., Baumgartner A., DeWitt J.C., Groves A., Mayr L., Weisman H.R. (2019). Resolving medulloblastoma cellular architecture by single-cell genomics. Nature.

[251] Misiak D., Hagemann S., Bell J.L., Busch B., Lederer M., Bley N., Schulte J.H., Hüttelmaier S. (2021). The MicroRNA landscape of MYCN-amplified neuroblastoma. Front. Oncol..

[252] Parodi F., Carosio R., Ragusa M., Di Pietro C., Maugeri M., Barbagallo D., Sallustio F., Allemanni G., Pistillo M.P., Casciano I. (2016). Epigenetic dysregulation in neuroblastoma: A tale of miRNAs and DNA methylation. Biochim. Biophys. Acta (BBA)-Gene Regul. Mech..

[253] Veeraraghavan V.P., Jayaraman S., Rengasamy G., Mony U., Ganapathy D.M., Geetha R.V., Sekar D. (2021). Deciphering the role of microRNAs in neuroblastoma. Molecules.

[254] Anelli L., Zagaria A., Specchia G., Musto P., Albano F. (2021). Dysregulation of miRNA in leukemia: Exploiting miRNA expression profiles as biomarkers. Int. J. Mol. Sci..

[255] Boldrin E., Gaffo E., Niedermayer A., Boer J.M., Zimmermann M., Weichenhan D., Claus R., Münch V., Sun Q., Enzenmüller S. (2021). MicroRNA-497/195 is tumor suppressive and cooperates with CDKN2A/B in pediatric acute lymphoblastic leukemia. Blood J. Am. Soc. Hematol..

[256] Gutierrez-Camino A., Garcia-Obregon S., Lopez-Lopez E., Astigarraga I., Garcia-Orad A. (2020). miRNA deregulation in childhood acute lymphoblastic leukemia: A systematic review. Epigenomics.

[257] Bo L., Wang Y., Li Y., Wurpel J.N., Huang Z., Chen Z.-S. (2023). The battlefield of chemotherapy in pediatric cancers. Cancers.

[258] Gareev I., Beylerli O., Liang Y., Xiang H., Liu C., Xu X., Yuan C., Ahmad A., Yang G. (2021). The role of MicroRNAs in therapeutic resistance of malignant primary brain tumors. Front. Cell Dev. Biol..

[259] Tang Z., Lu Y., Chen Y., Zhang J., Chen Z., Wang Q. (2021). Research progress of MicroRNA in chemotherapy resistance of osteosarcoma. Technol. Cancer Res. Treat..

[260] Messiaen J., Jacobs S.A., De Smet F. (2023). The tumor micro-environment in pediatric glioma: Friend or foe?. Front. Immunol..

[261] Xiao Y., Yu D. (2021). Tumor microenvironment as a therapeutic target in cancer. Pharmacol. Ther..

[262] Lieberman N.A., DeGolier K., Kovar H.M., Davis A., Hoglund V., Stevens J., Winter C., Deutsch G., Furlan S.N., Vitanza N.A. (2019). Characterization of the immune microenvironment of diffuse intrinsic pontine glioma: Implications for development of immunotherapy. Neuro-Oncology.

[263] van den Bent M.J., Geurts M., French P.J., Smits M., Capper D., Bromberg J.E., Chang S.M. (2023). Primary brain tumours in adults. Lancet.

[264] Wu K., Lin K., Li X., Yuan X., Xu P., Ni P., Xu D. (2020). Redefining tumor-associated macrophage subpopulations and functions in the tumor microenvironment. Front. Immunol..

[265] Patel R.R., Ramkissoon S.H., Ross J., Weintraub L. (2020). Tumor mutational burden and driver mutations: Characterizing the genomic landscape of pediatric brain tumors. Pediatr. Blood Cancer.

[266] Roux A., Pallud J., Saffroy R., Edjlali-Goujon M., Debily M.-A., Boddaert N., Sanson M., Puget S., Knafo S., Adam C. (2020). High-grade gliomas in adolescents and young adults highlight histomolecular differences from their adult and pediatric counterparts. Neuro-Oncology.

[267] Simon A.K., Hollander G.A., McMichael A. (2015). Evolution of the immune system in humans from infancy to old age. Proc. R. Soc. B Biol. Sci..

[268] Baumgarth N. (2021). The shaping of a B cell pool maximally responsive to infections. Annu. Rev. Immunol..

[269] Lam J.H., Smith F.L., Baumgarth N. (2020). B cell activation and response regulation during viral infections. Viral Immunol..

[270] Reina-Campos M., Scharping N.E., Goldrath A.W. (2021). CD8+ T cell metabolism in infection and cancer. Nat. Rev. Immunol..

[271] Van der Leun A.M., Thommen D.S., Schumacher T.N. (2020). CD8+ T cell states in human cancer: Insights from single-cell analysis. Nat. Rev. Cancer.

[272] Kobayashi M., Yoshimoto M. (2023). Multiple waves of fetal-derived immune cells constitute adult immune system. Immunol. Rev..

[273] Menon A.P., Moreno B., Meraviglia-Crivelli D., Nonatelli F., Villanueva H., Barainka M., Zheleva A., Van Santen H.M., Pastor F. (2023). Modulating T cell responses by targeting CD3. Cancers.

[274] Waldman A.D., Fritz J.M., Lenardo M.J. (2020). A guide to cancer immunotherapy: From T cell basic science to clinical practice. Nat. Rev. Immunol..

[275] Azevedo M.M., Pina-Vaz C., Baltazar F. (2020). Microbes and cancer: Friends or faux?. Int. J. Mol. Sci..

[276] Abad E., Graifer D., Lyakhovich A. (2020). DNA damage response and resistance of cancer stem cells. Cancer Lett..

[277] Ma Y., Chen T., Sun T., Dilimulati D., Xiao Y. (2024). The oncomicrobiome: New insights into microorganisms in cancer. Microb. Pathog..

[278] Bert S., Ward E.J., Nadkarni S. (2021). Neutrophils in pregnancy: New insights into innate and adaptive immune regulation. Immunology.

[279] Patel A.A., Ginhoux F., Yona S. (2021). Monocytes, macrophages, dendritic cells and neutrophils: An update on lifespan kinetics in health and disease. Immunology.

[280] True H., Blanton M., Sureshchandra S., Messaoudi I. (2022). Monocytes and macrophages in pregnancy: The good, the bad, and the ugly. Immunol. Rev..

[281] Wu Y., Hirschi K.K. (2021). Tissue-resident macrophage development and function. Front. Cell Dev. Biol..

[282] Pieren D.K., Boer M.C., de Wit J. (2022). The adaptive immune system in early life: The shift makes it count. Front. Immunol..

[283] Semmes E.C., Chen J.-L., Goswami R., Burt T.D., Permar S.R., Fouda G.G. (2021). Understanding early-life adaptive immunity to guide interventions for pediatric health. Front. Immunol..

[284] Hau P.M., Lung H.L., Wu M., Tsang C.M., Wong K.-L., Mak N.K., Lo K.W. (2020). Targeting Epstein-Barr virus in nasopharyngeal carcinoma. Front. Oncol..

[285] Jimenez O., Colli S., Garcia Lombardi M., Preciado M.V., De Matteo E., Chabay P. (2021). Epstein–Barr virus recruits PDL1-positive cells at the microenvironment in pediatric Hodgkin lymphoma. Cancer Immunol. Immunother..

[286] Wong Y., Meehan M.T., Burrows S.R., Doolan D.L., Miles J.J. (2022). Estimating the global burden of Epstein–Barr virus-related cancers. J. Cancer Res. Clin. Oncol..

[287] Frappier L. (2023). Epstein–Barr Virus is an Agent of Genomic Instability.

[288] Harper K.L., Harrington E.M., Hayward C., Anene C.A., Wongwiwat W., White R.E., Whitehouse A. (2024). Virus-modified paraspeckle-like condensates are hubs for viral RNA processing and their formation drives genomic instability. Nat. Commun..

[289] Ahye N., Bellizzi A., May D., Wollebo H.S. (2020). The role of the JC virus in central nervous system tumorigenesis. Int. J. Mol. Sci..

[290] Mayr L., Steinmaurer T., Weseslindtner L., Madlener S., Strassl R., Gojo J., Azizi A.A., Slavc I., Peyrl A. (2023). Viral infections in pediatric brain tumor patients treated with targeted therapies. Pediatr. Blood Cancer.

[291] Del Valle L., Gordon J., Enam S., Delbue S., Croul S., Abraham S., Radhakrishnan S., Assimakopoulou M., Katsetos C.D., Khalili K. (2002). Expression of human neurotropic polyomavirus JCV late gene product agnoprotein in human medulloblastoma. J. Natl. Cancer Inst..

[292] Kolaczkowska E., Kubes P. (2013). Neutrophil recruitment and function in health and inflammation. Nat. Rev. Immunol..

[293] Kumar R., Clermont G., Vodovotz Y., Chow C.C. (2004). The dynamics of acute inflammation. J. Theor. Biol..

[294] Medzhitov R. (2021). The spectrum of inflammatory responses. Science.

[295] Shi C., Pamer E.G. (2011). Monocyte recruitment during infection and inflammation. Nat. Rev. Immunol..

[296] Jiménez-Morales S., Aranda-Uribe I.S., Pérez-Amado C.J., Ramírez-Bello J., Hidalgo-Miranda A. (2021). Mechanisms of immunosuppressive tumor evasion: Focus on acute lymphoblastic leukemia. Front. Immunol..

[297] Morgenstern D.A., Anderson J. (2012). Inflammation: What role in pediatric cancer?. Pediatr. Blood Cancer.

[298] Segal B.H., Giridharan T., Suzuki S., Khan A.N.H., Zsiros E., Emmons T.R., Yaffe M.B., Gankema A.A., Hoogeboom M., Goetschalckx I. (2023). Neutrophil interactions with T cells, platelets, endothelial cells, and of course tumor cells. Immunol. Rev..

[299] Yee P.P., Li W. (2021). Tumor necrosis: A synergistic consequence of metabolic stress and inflammation. Bioessays.

[300] Bockmayr M., Klauschen F., Maire C.L., Rutkowski S., Westphal M., Lamszus K., Schüller U., Mohme M. (2019). Immunologic profiling of mutational and transcriptional subgroups in pediatric and adult high-grade gliomas. Cancer Immunol. Res..

[301] Bockmayr M., Mohme M., Klauschen F., Winkler B., Budczies J., Rutkowski S., Schüller U. (2018). Subgroup-specific immune and stromal microenvironment in medulloblastoma. Oncoimmunology.

[302] Chen Z., Hambardzumyan D. (2018). Immune microenvironment in glioblastoma subtypes. Front. Immunol..

[303] Grabovska Y., Mackay A., O’Hare P., Crosier S., Finetti M., Schwalbe E.C., Pickles J.C., Fairchild A.R., Avery A., Cockle J. (2020). Pediatric pan-central nervous system tumor analysis of immune-cell infiltration identifies correlates of antitumor immunity. Nat. Commun..

[304] Güç E., Pollard J.W. (2021). Redefining macrophage and neutrophil biology in the metastatic cascade. Immunity.

[305] Wang Y., Johnson K.C.C., Gatti-Mays M.E., Li Z. (2022). Emerging strategies in targeting tumor-resident myeloid cells for cancer immunotherapy. J. Hematol. Oncol..

[306] Doak G.R., Schwertfeger K.L., Wood D.K. (2018). Distant relations: Macrophage functions in the metastatic niche. Trends Cancer.

[307] Frederico S.C., Sharma N., Darling C., Taori S., Dubinsky A.C., Zhang X., Raphael I., Kohanbash G. (2024). Myeloid cells as potential targets for immunotherapy in pediatric gliomas. Front. Pediatr..

[308] Murdoch C., Muthana M., Coffelt S.B., Lewis C.E. (2008). The role of myeloid cells in the promotion of tumour angiogenesis. Nat. Rev. Cancer.

[309] Ochoa A.C., Zea A.H., Hernandez C., Rodriguez P.C. (2007). Arginase, prostaglandins, and myeloid-derived suppressor cells in renal cell carcinoma. Clin. Cancer Res..

[310] Kailayangiri S., Altvater B., Urban K., Meltzer J., Greune L., Farwick N., Jamitzky S., Rossig C. (2020). Evaluation of anti-Gr1 antibody for depletion of MDSC in preclinical NSG mouse models of pediatric sarcoma. Cancer Res..

[311] Perzolli A., Koedijk J.B., Zwaan C.M., Heidenreich O. (2024). Targeting the innate immune system in pediatric and adult AML. Leukemia.

[312] Thakur M.D., Franz C.J., Brennan L., Brouwer-Visser J., Tam R., Korski K., Koeppen H., Ziai J., Babitzki G., Ranchere-Vince D. (2022). Immune contexture of paediatric cancers. Eur. J. Cancer.

[313] Fujiwara N., Kobayashi K. (2005). Macrophages in inflammation. Curr. Drug Targets-Inflamm. Allergy.

[314] Motwani M.P., Gilroy D.W. (2015). Macrophage development and polarization in chronic inflammation. Semin Immunol.

[315] Lavin Y., Mortha A., Rahman A., Merad M. (2015). Regulation of macrophage development and function in peripheral tissues. Nat. Rev. Immunol..

[316] Chen Z., Feng X., Herting C.J., Garcia V.A., Nie K., Pong W.W., Rasmussen R., Dwivedi B., Seby S., Wolf S.A. (2017). Cellular and molecular identity of tumor-associated macrophages in glioblastoma. Cancer Res..

[317] Sidibe A., Ropraz P., Jemelin S., Emre Y., Poittevin M., Pocard M., Bradfield P.F., Imhof B.A. (2018). Angiogenic factor-driven inflammation promotes extravasation of human proangiogenic monocytes to tumours. Nat. Commun..

[318] Liu Y.-C., Zou X.-B., Chai Y.-F., Yao Y.-M. (2014). Macrophage polarization in inflammatory diseases. Int. J. Biol. Sci..

[319] Murray P.J. (2017). Macrophage polarization. Annu. Rev. Physiol..

[320] Ishina I.A., Zakharova M.Y., Kurbatskaia I.N., Mamedov A.E., Belogurov A.A., Gabibov A.G. (2023). MHC class II presentation in autoimmunity. Cells.

[321] Marrocco A., Ortiz L.A. (2022). Role of metabolic reprogramming in pro-inflammatory cytokine secretion from LPS or silica-activated macrophages. Front. Immunol..

[322] Dehne N., Mora J., Namgaladze D., Weigert A., Brüne B. (2017). Cancer cell and macrophage cross-talk in the tumor microenvironment. Curr. Opin. Pharmacol..

[323] Zhang J., Yuan X., Wang Y., Liu J., Li Z., Li S., Liu Y., Gong X., Sun Y., Wu W. (2022). Tumor-associated macrophages correlate with prognosis in medulloblastoma. Front. Oncol..

[324] Lin H., Wei S., Hurt E.M., Green M.D., Zhao L., Vatan L., Szeliga W., Herbst R., Harms P.W., Fecher L.A. (2018). Host expression of PD-L1 determines efficacy of PD-L1 pathway blockade–mediated tumor regression. J. Clin. Investig..

[325] Petty A.J., Dai R., Lapalombella R., Baiocchi R.A., Benson D.M., Li Z., Huang X., Yang Y. (2021). Hedgehog-induced PD-L1 on tumor-associated macrophages is critical for suppression of tumor-infiltrating CD8+ T cell function. JCI Insight.

[326] Aoki T., Hino M., Koh K., Kyushiki M., Kishimoto H., Arakawa Y., Hanada R., Kawashima H., Kurihara J., Shimojo N. (2016). Low frequency of programmed death ligand 1 expression in pediatric cancers. Pediatr. Blood Cancer.

[327] Pinto N., Park J.R., Murphy E., Yearley J., McClanahan T., Annamalai L., Hawkins D.S., Rudzinski E.R. (2017). Patterns of PD-1, PD-L1, and PD-L2 expression in pediatric solid tumors. Pediatr. Blood Cancer.

[328] van Dam L.S., de Zwart V.M., Meyer-Wentrup F.A. (2015). The role of programmed cell death-1 (PD-1) and its ligands in pediatric cancer. Pediatr. Blood Cancer.

[329] Cersosimo F., Lonardi S., Bernardini G., Telfer B., Mandelli G.E., Santucci A., Vermi W., Giurisato E. (2020). Tumor-associated macrophages in osteosarcoma: From mechanisms to therapy. Int. J. Mol. Sci..

[330] Dumars C., Ngyuen J.-M., Gaultier A., Lanel R., Corradini N., Gouin F., Heymann D., Heymann M.-F. (2016). Dysregulation of macrophage polarization is associated with the metastatic process in osteosarcoma. Oncotarget.

[331] Han Y., Guo W., Ren T., Huang Y., Wang S., Liu K., Zheng B., Yang K., Zhang H., Liang X. (2019). Tumor-associated macrophages promote lung metastasis and induce epithelial-mesenchymal transition in osteosarcoma by activating the COX-2/STAT3 axis. Cancer Lett..

[332] Liu K.X., Joshi S. (2020). “Re-educating” tumor associated macrophages as a novel immunotherapy strategy for neuroblastoma. Front. Immunol..

[333] Cabeza-Cabrerizo M., Cardoso A., Minutti C.M., Pereira da Costa M., Reis e Sousa C. (2021). Dendritic cells revisited. Annu. Rev. Immunol..

[334] Merad M., Sathe P., Helft J., Miller J., Mortha A. (2013). The dendritic cell lineage: Ontogeny and function of dendritic cells and their subsets in the steady state and the inflamed setting. Annu. Rev. Immunol..

[335] Wesa A.K., Galy A. (2001). IL-1β induces dendritic cells to produce IL-12. Int. Immunol..

[336] Guilliams M., Dutertre C.-A., Scott C.L., McGovern N., Sichien D., Chakarov S., Van Gassen S., Chen J., Poidinger M., De Prijck S. (2016). Unsupervised high-dimensional analysis aligns dendritic cells across tissues and species. Immunity.

[337] Segura E. (2022). Human dendritic cell subsets: An updated view of their ontogeny and functional specialization. Eur. J. Immunol..

[338] Albert M.L., Sauter B., Bhardwaj N. (1998). Dendritic cells acquire antigen from apoptotic cells and induce class I-restricted CTLs. Nature.

[339] Musella M., Galassi C., Manduca N., Sistigu A. (2021). The yin and yang of type I IFNs in cancer promotion and immune activation. Biology.

[340] Reizis B. (2019). Plasmacytoid dendritic cells: Development, regulation, and function. Immunity.

[341] Vakkila J., Jaffe R., Michelow M., Lotze M.T. (2006). Pediatric cancers are infiltrated predominantly by macrophages and contain a paucity of dendritic cells: A major nosologic difference with adult tumors. Clin. Cancer Res..

[342] Gassmann H., Schneider K., Evdokimova V., Ruzanov P., Schober S.J., Xue B., von Heyking K., Thiede M., Richter G.H., Pfaffl M.W. (2021). Ewing sarcoma-derived extracellular vesicles impair dendritic cell maturation and function. Cells.

[343] Melcher V., Kerl K. (2021). The growing relevance of immunoregulation in pediatric brain tumors. Cancers.

[344] Olsen H.E., Lynn G.M., Valdes P.A., Cerecedo Lopez C.D., Ishizuka A.S., Arnaout O., Bi W.L., Peruzzi P.P., Chiocca E.A., Friedman G.K. (2021). Therapeutic cancer vaccines for pediatric malignancies: Advances, challenges, and emerging technologies. Neuro-Oncol. Adv..

[345] Plantinga M., Lo Presti V., de Haar C.G., Dünnebach E., Madrigal A., Lindemans C.A., Boelens J.J., Nierkens S. (2020). Clinical grade production of wilms’ tumor-1 loaded cord blood-derived dendritic cells to prevent relapse in pediatric AML after cord blood transplantation. Front. Immunol..

[346] de Bruijn S., Anguille S., Verlooy J., Smits E.L., van Tendeloo V.F., De Laere M., Norga K., Berneman Z.N., Lion E. (2019). Dendritic cell-based and other vaccination strategies for pediatric cancer. Cancers.

[347] Van Acker H.H., Anguille S., Van Tendeloo V.F., Lion E. (2015). Empowering gamma delta T cells with antitumor immunity by dendritic cell-based immunotherapy. Oncoimmunology.

[348] Alzumaili B., Xu B., Spanheimer P.M., Tuttle R.M., Sherman E., Katabi N., Dogan S., Ganly I., Untch B.R., Ghossein R.A. (2020). Grading of medullary thyroid carcinoma on the basis of tumor necrosis and high mitotic rate is an independent predictor of poor outcome. Mod. Pathol..

[349] Moon S.W., Kim J.J., Jeong S.C., Kim Y.H., Han J.W. (2022). Clinical significance of tumor necrosis and viability in non-small cell lung cancer. J. Thorac. Dis..

[350] Karsch-Bluman A., Benny O. (2020). Necrosis in the tumor microenvironment and its role in cancer recurrence. Tumor Microenviron. Recent Adv..

[351] Land W.G. (2015). The role of damage-associated molecular patterns (DAMPs) in human diseases: Part II: DAMPs as diagnostics, prognostics and therapeutics in clinical medicine. Sultan Qaboos Univ. Med. J..

[352] Chirra M., Newton H.S., Gawali V.S., Wise-Draper T.M., Chimote A.A., Conforti L. (2022). How the potassium channel response of T lymphocytes to the tumor microenvironment shapes antitumor immunity. Cancers.

[353] Eil R., Vodnala S.K., Clever D., Klebanoff C.A., Sukumar M., Pan J.H., Palmer D.C., Gros A., Yamamoto T.N., Patel S.J. (2016). Ionic immune suppression within the tumour microenvironment limits T cell effector function. Nature.

[354] Bomken S., Davies B., Chong L., Cole M., Wood K.M., McDermott M., Tweddle D.A. (2011). Percentage tumor necrosis following chemotherapy in neuroblastoma correlates with MYCN status but not survival. Pediatr. Hematol. Oncol..

[355] Hanif I., Mahmoud H., Pui C.H. (1993). Avascular femoral head necrosis in pediatric cancer patients. Med. Pediatr. Oncol..

[356] Mersfelder R.B., Lwin C., Malik S., Badgett T.C., Chenard S.W., Rekulapelli A., Blette B.S., Lawrenz J.M., Borinstein S.C. (2024). Chemotherapy–Surgery Interval Effects on Tumor Necrosis and Outcome in Children and Young Adults With Osteosarcoma. Pediatr. Blood Cancer.

[357] Fiala E.M., Jayakumaran G., Mauguen A., Kennedy J.A., Bouvier N., Kemel Y., Fleischut M.H., Maio A., Salo-Mullen E.E., Sheehan M. (2021). Prospective pan-cancer germline testing using MSK-IMPACT informs clinical translation in 751 patients with pediatric solid tumors. Nat. Cancer.

[358] Hwang E.I., Sayour E.J., Flores C.T., Grant G., Wechsler-Reya R., Hoang-Minh L.B., Kieran M.W., Salcido J., Prins R.M., Figg J.W. (2022). The current landscape of immunotherapy for pediatric brain tumors. Nat. Cancer.

[359] Greten F.R., Grivennikov S.I. (2019). Inflammation and cancer: Triggers, mechanisms, and consequences. Immunity.

[360] Laha D., Grant R., Mishra P., Nilubol N. (2021). The role of tumor necrosis factor in manipulating the immunological response of tumor microenvironment. Front. Immunol..

[361] Freeman A.J., Kearney C.J., Silke J., Oliaro J. (2021). Unleashing TNF cytotoxicity to enhance cancer immunotherapy. Trends Immunol..

[362] Mori T., Sato Y., Miyamoto K., Kobayashi T., Shimizu T., Kanagawa H., Katsuyama E., Fujie A., Hao W., Tando T. (2014). TNFα promotes osteosarcoma progression by maintaining tumor cells in an undifferentiated state. Oncogene.

[363] Koo J., Hayashi M., Verneris M.R., Lee-Sherick A.B. (2020). Targeting tumor-associated macrophages in the pediatric sarcoma tumor microenvironment. Front. Oncol..

[364] Burger D., Molnarfi N., Gruaz L., Dayer J.-M. (2004). Differential induction of IL-1β and TNF by CD40 ligand or cellular contact with stimulated T cells depends on the maturation stage of human monocytes. J. Immunol..

[365] Dagenais M., Dupaul-Chicoine J., Douglas T., Champagne C., Morizot A., Saleh M. (2017). The Interleukin (IL)-1R1 pathway is a critical negative regulator of PyMT-mediated mammary tumorigenesis and pulmonary metastasis. Oncoimmunology.

[366] Mantovani A., Barajon I., Garlanda C. (2018). IL-1 and IL-1 regulatory pathways in cancer progression and therapy. Immunol. Rev..

[367] Zhang W., Borcherding N., Kolb R. (2020). IL-1 signaling in tumor microenvironment. Tumor Microenviron. Role Interleukins–Part A.

[368] Landuzzi L., Ruzzi F., Pellegrini E., Lollini P.-L., Scotlandi K., Manara M.C. (2024). IL-1 Family Members in Bone Sarcomas. Cells.

[369] Soker M., Çolpan L., Ece A., Devecioğlu C., Haspolat K. (2001). Serum levels of IL-1 beta, sIL-2R, IL-6, IL-8, and TNF-alpha in febrile children with cancer and neutropenia. Med. Oncol..

[370] Hirano T. (2021). IL-6 in inflammation, autoimmunity and cancer. Int. Immunol..

[371] Rašková M., Lacina L., Kejík Z., Venhauerová A., Skaličková M., Kolář M., Jakubek M., Rosel D., Smetana K., Brábek J. (2022). The role of IL-6 in cancer cell invasiveness and metastasis—Overview and therapeutic opportunities. Cells.

[372] Song L., Wang S., Fang T., Qiu X., Wang X., Zhou X., Morse M.A., Hobeika A., Wu W., Yang H. (2021). Changes in peripheral blood regulatory T cells and IL-6 and IL-10 levels predict response of pediatric medulloblastoma and germ cell tumors with residual or disseminated disease to craniospinal irradiation. Int. J. Radiat. Oncol. * Biol. * Phys..

[373] Stevens A.M., Miller J.M., Munoz J.O., Gaikwad A.S., Redell M.S. (2017). Interleukin-6 levels predict event-free survival in pediatric AML and suggest a mechanism of chemotherapy resistance. Blood Adv..

[374] Aliyu M., Zohora F.T., Anka A.U., Ali K., Maleknia S., Saffarioun M., Azizi G. (2022). Interleukin-6 cytokine: An overview of the immune regulation, immune dysregulation, and therapeutic approach. Int. Immunopharmacol..

[375] Sims N.A. (2021). Influences of the IL-6 cytokine family on bone structure and function. Cytokine.

[376] Chua L.L., Rajasuriar R., Azanan M.S., Abdullah N.K., Tang M.S., Lee S.C., Woo Y.L., Lim Y.A.L., Ariffin H., Loke P.n. (2017). Reduced microbial diversity in adult survivors of childhood acute lymphoblastic leukemia and microbial associations with increased immune activation. Microbiome.

[377] Masetti R., Muratore E., Leardini D., Zama D., Turroni S., Brigidi P., Esposito S., Pession A. (2021). Gut microbiome in pediatric acute leukemia: From predisposition to cure. Blood Adv..

[378] Vahabnezhad E., Mochon A.B., Wozniak L.J., Ziring D.A. (2013). Lactobacillus bacteremia associated with probiotic use in a pediatric patient with ulcerative colitis. J. Clin. Gastroenterol..

[379] Liu B., Huang Y., Sun Y., Zhang J., Yao Y., Shen Z., Xiang D., He A. (2016). Prognostic value of inflammation-based scores in patients with osteosarcoma. Sci. Rep..

[380] Brahmer J., Reckamp K.L., Baas P., Crinò L., Eberhardt W.E., Poddubskaya E., Antonia S., Pluzanski A., Vokes E.E., Holgado E. (2015). Nivolumab versus docetaxel in advanced squamous-cell non–small-cell lung cancer. N. Engl. J. Med..

[381] Rosenberg J.E., Hoffman-Censits J., Powles T., Van Der Heijden M.S., Balar A.V., Necchi A., Dawson N., O’Donnell P.H., Balmanoukian A., Loriot Y. (2016). Atezolizumab in patients with locally advanced and metastatic urothelial carcinoma who have progressed following treatment with platinum-based chemotherapy: A single-arm, multicentre, phase 2 trial. Lancet.

[382] Geoerger B., Kang H.J., Yalon-Oren M., Marshall L.V., Vezina C., Pappo A., Laetsch T.W., Petrilli A.S., Ebinger M., Toporski J. (2020). Pembrolizumab in paediatric patients with advanced melanoma or a PD-L1-positive, advanced, relapsed, or refractory solid tumour or lymphoma (KEYNOTE-051): Interim analysis of an open-label, single-arm, phase 1–2 trial. Lancet Oncol..

[383] Roemer M.G., Advani R.H., Ligon A.H., Natkunam Y., Redd R.A., Homer H., Connelly C.F., Sun H.H., Daadi S.E., Freeman G.J. (2016). PD-L1 and PD-L2 Genetic Alterations Define Classical Hodgkin Lymphoma and Predict Outcome. J. Clin. Oncol..

[384] Long A.H., Morgenstern D.A., Leruste A., Bourdeaut F., Davis K.L. (2022). Checkpoint Immunotherapy in Pediatrics: Here, Gone, and Back Again. In American Society of Clinical Oncology Educational book.

[385] Melaiu O., Lucarini V., Giovannoni R., Fruci D., Gemignani F. (2022). News on immune checkpoint inhibitors as immunotherapy strategies in adult and pediatric solid tumors. Seminars in Cancer Biology.

[386] Merchant M.S., Wright M., Baird K., Wexler L.H., Rodriguez-Galindo C., Bernstein D., Delbrook C., Lodish M., Bishop R., Wolchok J.D. (2016). Phase I Clinical Trial of Ipilimumab in Pediatric Patients with Advanced Solid Tumors. Clin. Cancer Res..

[387] Das A., Tabori U., Sambira Nahum L.C., Collins N.B., Deyell R., Dvir R., Faure-Conter C., Hassall T.E., Minturn J.E., Edwards M. (2023). Efficacy of Nivolumab in Pediatric Cancers with High Mutation Burden and Mismatch Repair Deficiency. Clin. Cancer Res..

[388] Dunkel I.J., Doz F., Foreman N.K., Hargrave D., Lassaletta A., Andre N., Hansford J.R., Hassall T., Eyrich M., Gururangan S. (2023). Nivolumab with or without ipilimumab in pediatric patients with high-grade CNS malignancies: Safety, efficacy, biomarker, and pharmacokinetics-CheckMate 908. Neuro-Oncology.

[389] Kaneda D., Iehara T., Kikuchi K., Sugimoto Y., Nakagawa N., Yagyu S., Miyachi M., Konishi E., Sakai T., Hosoi H. (2022). The histone deacetylase inhibitor OBP-801 has in vitro/in vivo anti-neuroblastoma activity. Pediatr. Int..

[390] Nawar N., Bukhari S., Adile A.A., Suk Y., Manaswiyoungkul P., Toutah K., Olaoye O.O., Raouf Y.S., Sedighi A., Garcha H.K. (2022). Discovery of HDAC6-selective inhibitor NN-390 with in vitro efficacy in group 3 medulloblastoma. J. Med. Chem..

[391] Chilamakuri R., Agarwal S. (2022). Dual targeting of PI3K and HDAC by CUDC-907 inhibits pediatric neuroblastoma growth. Cancers.

[392] Chang W.I., Honeyman J.N., Zhang J., Lin C., Sharma A., Zhou L., Oliveira J., Tapinos N., Lulla R.R., Prabhu V.V. (2023). Novel combination of imipridones and histone deacetylase inhibitors demonstrate cytotoxic effect through integrated stress response in pediatric solid tumors. Am. J. Cancer Res..

